# Multiple novel prostate cancer susceptibility signals identified by fine-mapping of known risk loci among Europeans

**DOI:** 10.1093/hmg/ddv203

**Published:** 2015-05-29

**Authors:** Ali Amin Al Olama, Tokhir Dadaev, Dennis J. Hazelett, Qiuyan Li, Daniel Leongamornlert, Edward J. Saunders, Sarah Stephens, Clara Cieza-Borrella, Ian Whitmore, Sara Benlloch Garcia, Graham G. Giles, Melissa C. Southey, Liesel Fitzgerald, Henrik Gronberg, Fredrik Wiklund, Markus Aly, Brian E. Henderson, Fredrick Schumacher, Christopher A. Haiman, Johanna Schleutker, Tiina Wahlfors, Teuvo L. Tammela, Børge G. Nordestgaard, Tim J. Key, Ruth C. Travis, David E. Neal, Jenny L. Donovan, Freddie C. Hamdy, Paul Pharoah, Nora Pashayan, Kay-Tee Khaw, Janet L. Stanford, Stephen N. Thibodeau, Shannon K. Mcdonnell, Daniel J. Schaid, Christiane Maier, Walther Vogel, Manuel Luedeke, Kathleen Herkommer, Adam S. Kibel, Cezary Cybulski, Dominika Wokołorczyk, Wojciech Kluzniak, Lisa Cannon-Albright, Hermann Brenner, Katja Butterbach, Volker Arndt, Jong Y. Park, Thomas Sellers, Hui-Yi Lin, Chavdar Slavov, Radka Kaneva, Vanio Mitev, Jyotsna Batra, Judith A. Clements, Amanda Spurdle, Manuel R. Teixeira, Paula Paulo, Sofia Maia, Hardev Pandha, Agnieszka Michael, Andrzej Kierzek, Koveela Govindasami, Michelle Guy, Artitaya Lophatonanon, Kenneth Muir, Ana Viñuela, Andrew A. Brown, The PRACTICAL Consortium, COGS-CRUK GWAS-ELLIPSE (Part of GAME-ON) Initiative, The Australian Prostate Cancer BioResource, The UK Genetic Prostate Cancer Study Collaborators, The UK ProtecT Study Collaborators, Mathew Freedman, David V. Conti, Douglas Easton, Gerhard A. Coetzee, Rosalind A. Eeles, Zsofia Kote-Jarai

**Affiliations:** 1 Centre for Cancer Genetic Epidemiology, Department of Public Health and Primary Care, Strangeways Research Laboratory,; 2 Department of Oncology, Addenbrooke's Hospital,; 3 Centre for Cancer Genetic Epidemiology, Department of Oncology, Strangeways Laboratory,; 4 Clinical Gerontology Unit, University of Cambridge, Cambridge, UK,; 5 Division of Genetics and Epidemiology, The Institute of Cancer Research & Royal Marsden NHS Foundation Trust, London, UK,; 6 Department of Urology, Norris Comprehensive Cancer Center, Keck School of Medicine, USC, Los Angeles, CA, USA,; 7 Department of Preventive Medicine, Norris Comprehensive Cancer Center, Keck School of Medicine, USC, Los Angeles, CA, USA,; 8 Medical College, Xiamen University, Xiamen, China,; 9 Cancer Epidemiology Centre, The Cancer Council Victoria, Melbourne, VIC, Australia,; 10 Centre for Epidemiology and Biostatistics, Melbourne School of Population and Global Health,; 11 Genetic Epidemiology Laboratory, Department of Pathology, The University of Melbourne, Parkville, VIC, Australia,; 12 Tissupath Pty Ltd., Melbourne, VIC, Australia,; 13 Department of Medical Epidemiology and Biostatistics, Karolinska Institute, Stockholm, Sweden,; 14 Department of Clinical Sciences, Danderyds Hospital, Stockholm, Sweden,; 15 Department of Medical Biochemistry and Genetics Institute of Biomedicine, University of Turku, Turku, Finland,; 16 BioMediTech, University of Tampere and FimLab Laboratories, Tampere, Finland,; 17 Department of Urology, Tampere University Hospital and Medical School, University of Tampere, Tampere, Finland,; 18 Department of Clinical Biochemistry, Herlev Hospital, Copenhagen University Hospital, Herlev, Denmark,; 19 Faculty of Health and Medical Sciences, University of Copenhagen, Copenhagen, Denmark,; 20 Cancer Epidemiology, Nuffield Department of Population Health,; 21 Nuffield Department of Surgical Sciences, University of Oxford, Oxford, UK,; 22 Faculty of Medical Science, John Radcliffe Hospital, University of Oxford, Oxford, UK,; 23 Cancer Research UK Cambridge Research Institute, Li Ka Shing Centre, Cambridge, UK,; 24 School of Social and Community Medicine, University of Bristol, Bristol, UK,; 25 Department of Applied Health Research, University College London, London, UK,; 26 Division of Public Health Sciences, Fred Hutchinson Cancer Research Center, Seattle, WA, USA,; 27 Department of Epidemiology, School of Public Health, University of Washington, Seattle, WA, USA,; 28 Mayo Clinic, Rochester, MN, USA,; 29 Department of Urology, University Hospital Ulm, Ulm, Germany,; 30 Institute of Human Genetics, University of Ulm, Ulm, Germany,; 31 Department of Urology, Klinikum rechts der Isar der Technischen Universitaet Muenchen, Munich, Germany,; 32 Division of Urologic Surgery, Brigham and Women’s Hospital, Dana-Farber Cancer Institute, Boston, USA,; 33 International Hereditary Cancer Center, Department of Genetics and Pathology, Pomeranian Medical University, Szczecin, Poland,; 34 Division of Genetic Epidemiology, Department of Medicine, University of Utah School of Medicine, Salt Lake City, UT, USA,; 35 George E. Wahlen Department of Veterans Affairs Medical Center, Salt Lake City, UT, USA,; 36 Division of Clinical Epidemiology and Aging Research, German Cancer Research Center (DKFZ), Heidelberg, Germany,; 37 German Cancer Consortium (DKTK), Heidelberg, Germany,; 38 Department of Cancer Epidemiology, Moffitt Cancer Center, Tampa, FL, USA,; 39 Biostatistics Program, Moffitt Cancer Center, Tampa, FL, USA,; 40 Department of Urology and Alexandrovska University Hospital, Medical University, Sofia, Bulgaria,; 41 Department of Medical Chemistry and Biochemistry, Molecular Medicine Center, Medical University, Sofia, Bulgaria,; 42 Australian Prostate Cancer Research Centre-Qld, Institute of Health and Biomedical Innovation and School of Biomedical Science, Queensland University of Technology, Brisbane, Australia,; 43 Molecular Cancer Epidemiology Laboratory, Queensland Institute of Medical Research, Brisbane, Australia,; 44 Department of Genetics, Portuguese Oncology Institute, Porto, Portugal,; 45 Biomedical Sciences Institute (ICBAS), University of Porto, Porto, Portugal,; 46 The University of Surrey, Guildford, Surrey, UK,; 47 Institute of Population Health, University of Manchester, Manchester, UK,; 48 Warwick Medical School, University of Warwick, Coventry, UK,; 49 Department of Twin Research & Genetic Epidemiology, King's College London, London, UK,; 50 NORMENT, KG Jebsen Centre for Psychosis Research, Institute of Clinical Medicine, University of Oslo, Oslo, Norway,; 51 Department of Genetic Medicine and Development, University of Geneva, Switzerland and; 52 Dana Farber Cancer Institute, Boston, MA, USA

## Abstract

Genome-wide association studies (GWAS) have identified numerous common prostate cancer (PrCa) susceptibility loci. We have fine-mapped 64 GWAS regions known at the conclusion of the iCOGS study using large-scale genotyping and imputation in 25 723 PrCa cases and 26 274 controls of European ancestry. We detected evidence for multiple independent signals at 16 regions, 12 of which contained additional newly identified significant associations. A single signal comprising a spectrum of correlated variation was observed at 39 regions; 35 of which are now described by a novel more significantly associated lead SNP, while the originally reported variant remained as the lead SNP only in 4 regions. We also confirmed two association signals in Europeans that had been previously reported only in East-Asian GWAS. Based on statistical evidence and linkage disequilibrium (LD) structure, we have curated and narrowed down the list of the most likely candidate causal variants for each region. Functional annotation using data from ENCODE filtered for PrCa cell lines and eQTL analysis demonstrated significant enrichment for overlap with bio-features within this set. By incorporating the novel risk variants identified here alongside the refined data for existing association signals, we estimate that these loci now explain ∼38.9% of the familial relative risk of PrCa, an 8.9% improvement over the previously reported GWAS tag SNPs. This suggests that a significant fraction of the heritability of PrCa may have been hidden during the discovery phase of GWAS, in particular due to the presence of multiple independent signals within the same region.

## Introduction

Prostate cancer (PrCa) is one of the most commonly diagnosed cancers and leading causes of cancer-related deaths for men in developed countries. An increased incidence of PrCa among first-degree relatives of patients, together with results from twin studies, provides strong evidence for a heritable component to PrCa (1). In recent years, many studies have sought to identify genetic variants that predispose towards the development of PrCa. Candidate gene studies have demonstrated that rare (minor allele frequency, MAF < 1%) loss-of-function variants in DNA repair genes, in particular *BRCA2*, as well as a recurrent missense variant in *HOXB13* confer moderately increased disease risks; however, these explain only a limited fraction of the overall heritability (2,3). In addition to these rare, higher risk mutations, ∼100 common, low-penetrance variants have currently been identified through GWAS. These variants confer only modest increases in risk individually, but appear to combine multiplicatively thereby exerting a more substantial effect that is currently estimated to explain 33% of the familial relative risk (FRR) of the disease (4).

The specific low penetrance variants identified in GWAS are generally unlikely themselves to be causative for PrCa, since they are typically correlated with many other variants, one or more of which is functionally related to the disease. Fine-mapping studies are therefore performed to enable a more thorough evaluation of variation in associated regions, in order to narrow down the number of potential causal variants for subsequent evaluation and validation through functional assays. In addition, it has become clear that a small number of regions associated with many traits harbor multiple independent association signals (a classic example of which is the Chr8q24 region centromeric to *MYC*, which is associated with many forms of cancer including PrCa). However, in most cases, it is unclear whether these independent signals modulate risk through a common or separate functional mechanism, since the causal variants themselves remain unresolved. A key first step towards identifying the precise causal variants and functional mechanisms that confer risk is to comprehensively evaluate the evidence for association for non-genotyped potentially relevant variants within the region, to refine the original GWAS signal. In principle, re-sequencing associated regions in large case–control series would provide the most thorough data, but this approach is currently prohibitively expensive for routine application. However, since GWAS signals are expected to be predominantly driven by relatively common variants, large-scale genotyping together with imputation provides a cost-effective approach to evaluate the majority of likely causal variants. To date, only a small number of PrCa susceptibility loci have been fine-mapped. In analyses conducted by the PRACTICAL Consortium, for which the largest set of PrCa samples and genotype data are available, we previously identified for the *KLK3* locus at Chr19q13 a more strongly associated missense coding variant that has been demonstrated to alter protein function (5), and at two regions, Chr8q24 and *TERT* at Chr5p15, fine-mapping demonstrated the presence of multiple independent risk variants (6,7). In this study, we have fine-mapped, functionally annotated and curated a set of the most promising candidate susceptibility variants for all PrCa susceptibility regions published by the end of the iCOGS genotyping project, aside from the three that we had previously analyzed individually.

## Results

We have fine-mapped 64 known PrCa regions through a combination of genotyping and imputation. Region boundaries for this analysis were defined as 500 kb either side of any known PrCa associated GWAS SNPs; where such regions overlapped, they were merged to form a single larger region (extended boundaries were employed at regions Chr3p12, Chr4q22, Chr8p21, Chr11q13 and Chr17q12). We used genotype data for 25 723 cases and 26 274 controls of European ancestry from two UK GWAS studies and from the 32 studies in the PRACTICAL Consortium genotyped using the iCOGS array. After imputation to a 1000 Genomes reference panel, data were available for 283 910 SNPs across these 64 regions. For 23 of the 64 regions the iCOGS array contained a dense panel of markers that included almost all variants correlated with the original GWAS hit, thereby facilitating particularly high-resolution interrogation of these loci.

In this fine-mapping study, 15 previously reported PrCa susceptibility variants did not replicate at genome-wide significance (*P* < 5 × 10^−8^). For four of these variants, the association with PrCa had previously been reported only in East Asian populations (rs1938781, rs2252004 (8) and rs9600079 (9) in Japanese and rs103294 (10) in Chinese individuals). We found no evidence suggestive of association with PrCa at any of these regions in individuals of European ancestry (*P* > 0.4), which may indicate that these variants modulate risk through a mechanism predominate among individuals with specific genetic backgrounds, or alternatively require further confirmation in Europeans through additional larger studies. For another variant that did not replicate at genome-wide significance in this study, rs1571801, the previously reported association with PrCa achieved statistical significance only in relation to aggressive disease (11). In our data, the most strongly associated correlated variant within this region showed some tendency towards association with PrCa although remained non-significant (rs200543781, OR = 1.36, *P* = 5.1 × 10^−4^); this could reflect the fact that our sample panel was not enriched for aggressive disease. A further four regions had previously been identified in studies by the PRACTICAL Consortium; however, in each case, a larger sample size was available in the original study or consequent replication set than for this fine-mapping analysis. For these four SNPs (rs6869841, rs2427345 and rs11902236 published in Eeles *et al.* (12) and rs6763931 in Kote-Jarai *et al.* (13)), *P*-values in this analysis were close to genome-wide significance (Supplementary Material, Table S1), and the failure to replicate at genome-wide significance most likely reflects the smaller sample size. For the remaining six regions where the original index SNP did not reach genome-wide significance, our recent meta-analysis that included additional datasets (35 093 cases and 34 599 controls, Amin Al Olama *et al*. (4)) observed associations at genome-wide significance, and therefore these regions were included in this analysis. Of the 55 regions in our final analysis (after excluding the four non-European and five European regions which did not reach genome-wide significance in this study or the meta-analysis), after stepwise logistic regression, 39 could be categorized as ‘simple regions' defined by a single association signal (Table 1) and 16 as ‘complex regions', each of which contained more than one independent association signal (Table 2).

**Table 1.** Simple regions: fine-mapped regions where a single signal remained following stepwise logistic regression.

Chr (region)	Previous hit	Region boundary (Hg19)	New index SNP (*P*-value)	Alleles (ref/alt)—OR (95% CI)	Imputation quality *r^2^*-LD *r^2^* with previous hit	Number of best candidate SNPs (number overlapping bio-features) [eQTLs]
1q21 (1_1)	rs1218582	154334253–155332994	rs4845695 (2.7 × 10^−8^)	A/G – 0.93 (0.90–0.95)	0.97–0.5	78 (26)
1q32 (1_2)	rs4245739	203997926–204997638	rs199774366 (5.4 × 10^−11^)	A/AAC—0.91 (0.88–0.94)	0.96–0.91	78 (22)
2p24 (2_2)	rs13385191^#^	20388443–21388224	rs9306895 (6.5 × 10^−10^)	T/C—0.92 (0.90–0.95)	0.92–0.58	5 (1)
2p21^a^ (2_3)	rs1465618	42985311–43984987	rs7591218 (3.8 × 10^−10^)	A/G—1.09 (1.06–1.12)	0.99–0.34	8 (5)
2p11 (2_5)	rs10187424	85294918–86293829	rs2028900 (3.1 × 10^−16^)	T/C—0.90 (0.87–0.92)	0.90–0.83	42 (16)
2q37 (2_7)	rs2292884	237943293–238943056	rs11891348 (2.1 × 10^−8^)	T/G—0.92 (0.89–0.95)	0.94–0.40	36 (3) *MLPH*
3q13 (3_2)	rs7611694	112775825–113775563	rs6769767 (1.3 × 10^−15^)	A/G—0.90 (0.87–0.93)	0.94–0.83	14 (3)
4q13 (4_1)	rs1894292	73692431–74691942	rs1894292 (1.4 × 10^−11^)	A/G—0.92 (0.89–0.94)	1–n/a	11 (1)
4q24^a^ (4_3)	rs7679673	105561718–106561058	rs34480284 (8.0 × 10^−29^)	T/TA—1.16 (1.13–1.18)	0.99–0. 98	13 (2)
5p15 (5_2)	rs12653946^#^	1396112–2395482	rs10866527 (1.1 × 10^−8^)	T/C—1.08 (1.06–1.11)	0.76–0.75	6 (3) *IRX4*
5p12 (5_3)	rs2121875	43687710–44686471	rs1482679 (2.7 × 10^−9^)	A/G—0.92 (0.89–0.95)	0.81–0.91	91 (0)
6p21 (6_1)	rs130067^&^	30548676–31548176	rs2596546 (1.0 × 10^−9^)	A/G—1.09 (1.06–1.12)	0.97–0.02	1 (0)
6p21 (6_2)	rs3096702	31711572–32711433	rs115306967^b^ (6.4 × 10^−7^)	C/G—0.93 (0.90–0.96)	0.97–0.06	42 (9)
6p21 (6_3)	rs1983891^#^	41036770–42036395	rs6458228 (4.7 × 10^−8^)	A/C—1.08 (1.05–1.11)	0.92–0.90	33 (12)
6p21 (6_4)	rs2273669	108785991–109784474	rs12209480^b^ (8.9 × 10^−7^)	A/G—1.12 (1.07–1.16)	0.88–0.39	4 (0)
6q22 (6_5)	rs339331^#^	116827662–117827493	rs200820108 (2.0 × 10^−10^)	A/ATT—0.90 (0.87–0.94)	0.91–0.64	34 (4)
6q21^a^ (6_6)	rs1933488	152941182–153941032	rs3968480^b^ (8.9 × 10^−7^)	A/G—0.90 (0.87–0.92)	0.97–0.92	54 (4) *RGS17*
7p15 (7_1)	rs12155172	20529474–21529302	rs10713532 (4.1 × 10^−14^)	T/TG—0.89 (0.86–0.92)	0.99–0.99	3 (1)
7q21^a^ (7_3)	rs6465657	97316451–98316171	rs6965016 (1.5 × 10^−20^)	A/C—0.89 (0.86–0.91)	1–0.99	54 (15)
8p21 (8_2)	rs11135910	25392758–26391862	rs6984769^b^ (1.9 × 10^−7^)	T/C—1.10 (1.06–1.13)	0.88–0.88	30 (5) *EBF2*
9q31 (9_1)	rs817826^&^	109656878–110656064	rs1771718 (1.6 × 10^−8^)	T/C—0.92 (0.89–0.95)	0.83–0.03	47 (31)
10q11^a^ (10_1)	rs10993994	51049548–52049482	rs10993994 (6.2 × 10^−72^)	T/C—1.26 (1.24–1.29)	1–n/a	1 (1)
10q24 (10_2)	rs3850699	103914882–104913940	rs34032774 (1.4 × 10^−8^)	CT/C—1.09 (1.06–1.11)	0.91–0.94	33 (10) *C10orf32*/ *TMEM180*/*AS3MT*
10q26^a^ (10_4)	rs4962416	126447545–127446195	rs67609008^b^ (6.0 × 10^−5^)	T/C—0.94 (0.91–0.97)	0.97–0.42	10 (4) *CTBP2*
11p15^a^ (11_1)	rs7127900	1733857–2733077	rs11043143 (2.3 × 10^−42^)	T/C—1.24 (1.21–1.27)	1–0.97	26 (16) *ASCL2*
11q22 (11_4)	rs11568818	101902241–102901389	rs11568818 (2.0 × 10^−10^)	T/C—1.09 (1.06–1.12)	1– n/a	2 (2) *MMP7*
12q13 (12_1)	rs10875943	49176582–50175686	rs10875943 (4.2 × 10^−12^)	T/C—0.91 (0.88–0.93)	1–n/a	6 (3)
12q13^a^ (12_2)	rs902774	52774067–53773299	rs73110471 (7.4 × 10^−19^)	A/G—1.18 (1.14–1.22)	0.98–0.49	28 (6)
14q22 (14_1)	rs8008270	52872457–53872104	rs62003539 (4.5 × 10^−13^)	T/C—1.15 (1.11–1.19)	0.97–0.66	6 (1)
17p13 (17_1)	rs684232	119162–1118931	rs461251 (6.2 × 10^−15^)	A/G—0.90 (0.88–0.93)	1–0.90	13 (5) *VS53*/*FAM57A*
17q24^a^ (17_4)	rs1859962	68609232–69608508	rs8072735 (2.4 × 10^−50^)	T/C—1.21 (1.19–1.24)	0.99–0.76	19 (9)
18q23 (18_1)	rs7241993	76177342–77176537	rs9959454 (3.9 × 10^−9^)	A/G—1.09 (1.06–1.12)	1–0.81	12 (3)
19q13^a^ (19_1)	rs8102476	38235839–39235539	rs12610267 (3.7 × 10^−13^)	A/G—1.10 (1.07–1.12)	0.95–0.81	15 (6) *CATSPERG*
19q13^a^ (19_2)	rs11672691	41485821–42485578	rs74738513 (2.5 × 10^−12^)	A/T—0.90 (0.87–0.93)	0.85–0.98	8 (4)
20q13 (20_2)	rs6062509	61863226–62862439	rs1058319 (1.4 × 10^−14^)	T/C—0.84 (0.80–0.88)	0.85–0.22	1 (1)
22q13 (22_1)	rs9623117	39952275–40952051	rs11704314^b^ (1.7 × 10^−6^)	A/G—0.92 (0.88–0.95)	0.86–0.06	2 (2)
Xp22 (23_1)	rs2405942	9314154–10314083	rs2405943 (3.1 × 10^−11^)	T/C—0.93 (0.91–0.95)	1–0.90	10 (0)
Xp11^a^ (23_2)	rs5945619	50742323–51741595	rs1541241 (8.0 × 10^−33^)	T/G—1.12 (1.10–1.14)	1–0.95	93 (0)
Xq12 (23_3)	rs5919432	66522881–67520014	rs4446868 (3.6 × 10^−8^)	T/G—0.93 (0.90–0.96)	0.77–0.55	46 (0)

Fine-mapping identified a single, more strongly associated variant at 39 regions. Imputation quality and correlation (LD) between these and the original GWAS signal are indicated. We confirmed association with PrCa in populations of European ancestry for one variant originally identified in **^#^**Japanese and one variant reported for ^&^Chinese individuals, which had not been reported for Europeans previously. Four variants previously reported for Japanese or Chinese ancestry populations showed no evidence for replication in Europeans in this analysis (see Supplementary Material, Table S1). The *KLK* region at Chr19 was not included here as this region had previously been fine-mapped individually (5). Best candidate SNPs are variants correlated at *r^2^* > 0.7 with the lead variant describing an association, and with odds of association ≥1/1000 relative to the lead variant for the region.

eQTL data indicate statistically significant correlation between the new index SNP and gene expression in 145 prostate tumor samples from the TCGA dataset.

^a^These regions were densely genotyped on the iCOGS chip to fine-map PrCa associations known at the time of design.

^b^The top SNP in these six regions did not achieve genome-wide significance in iCOGS/UKGWAS but was significant in a larger meta-analysis study (4).

**Table 2.** Complex regions: multiple independent associations were identified in 16 regions following stepwise logistic regression.

Chr (region)	Previous hit(s)	Region boundaries (Hg19)	Best signal in meta-analysis (*P*-value)	Independent lead SNPs	Alleles (ref/alt)—OR (95%) in the final model	Imputation quality *r^2^*: LD (*r^2^*) with original index SNP (first/second original SNP): LD (*r^2^*) with new best signal	Number of best candidate SNPs (number overlapping bio-features) [eQTLs]
2p15^a^ (2_4)	rs721048	62631731–63631731	rs58235267 (3.9 × 10^−26^)	rs58235267 (3.1 × 10^−21^)	C/G—0.88 (0.85–0.90)	0.88: 0.12: n/a	1 (1)
				rs901532 (3.5 × 10^−6)^	T/C—1.10 (1.06–1.14)	0.99: 0.07: 0.03	3 (1) *EHBP1*
2q31^a^ (2_6)	rs12621278	172811553–173811553	rs13410475 (8.3 × 10^−26^)	rs13410475 (2.1 × 10^−15^)	A/C—0.78 (0.72–0.84)	0.97: 1: n/a	74 (18)
				rs12151618 (3.36 × 10^−7^)	T/C—0.92 (0.88–0.95)	0.93: 0.09: 0.09	4 (0)
2q37 (2_8)	rs3771570	241882864–242882864	rs111770284 (1.6 × 10^−13^)	rs111770284 (3.03 × 10^−12^)	T/C—1.13 (1.10–1.17)	0.85: 0.03: n/a	4 (3)
				rs183997311 (8.63 × 10^−8^)	A/G—0.67 (0.53–0.82)	0.58: 0.002: 0.002	7 (2)
3p12^a^ (3_1)	rs2660753 rs2055109	86610674–87967332	rs2088396 (6.5 × 10^−23^)	rs2088396 (5.75 × 10^−15^)	C/G—0.90 (0.88–0.93)	0.99: (0.03/0.01): n/a	17 (0)
				rs143351723 (2.88 × 10^−10^)	C/G—0.85 (0.80–0.90)	0.97: (0.36: 0.03): 0.06	51 (5)
				rs114278123 (1.75 × 10^−5^)	A/G—0.86 (0.79–0.93)	0.99: (0/0): 0.008	7 (2)
3q21^a^ (3_3)	rs10934853	127710474–128284711	rs2811485 (1.1 × 10^−15^)	rs2811485 (4.76 × 10^−15^)	T/G—1.12 (109–1.15)	0.99: 0.81: n/a	78 (10)
				rs56325233 (2.21 × 10^−6^)	C/G—1.07 (1.04–1.10)	0.94: 0.003: 0.0007	28 (0)
3q26^a^ (3_5)	rs10936632	169689793–170395852	rs78416326 (6.4 × 10^−25^)	rs78416326 (1.8 × 10^−28^)	C/G—0.83 (0.79–0.86)	0.80: 0.13: n/a	2 (0)
				rs11288195 (7.49 × 10^−9^)	A/AG—1.12 (1.08–1.16)	0.98: 0.122: 0.028	2 (1)
4q22^a^ (4_2)	rs12500426 rs17021918	95018784–95600782	rs7682375 (4.0 × 10^−19^)	rs7682375 (1.10 × 10^−6^)	A/T—1.09 (1.05–1.12)	1: (0.38/0.69): n/a	20 (6) *BMPR1B*
				rs6853490 (4.78 × 10^−6^)	A/G—0.93 (0.90–0.96)	0.95: (0.76/0.24): 0.30	7 (3) *BMPR1B*
6p25^a^ (6_7)	rs9364554	160374745–161323288	rs4646284 (3.2 × 10^−47^)	rs4646284 (5.40 × 10^−38^)	T/TG—0.81 (0.78–0.84)	0.77: 0.04: n/a	1 (1)
				rs2063347 (4.58 × 10^−7^)	A/G—1.07 (1.04–1.10)	1: 0.58: 0.022	18 (8)
7p15^a^ (7_2)	rs10486567	27550633–28102614	rs10486567 (7.3 × 10^−22^)	rs10486567 (2.62 × 10^−15^)	A/G—0.88 (0.85–0.91)	1: 1: n/a	22 (7)
				rs200362064 (9.04 × 10^−6^)	T/TGATA—0.94 (0.92–0.97)	0.97: 0.034: 0.034	15 (6)
8p21^a^ (8_1)	rs2928679 rs1512268	23100674–23548146	rs13272392 (1.3 × 10^−26^)	rs13272392 (8.7 × 10^−31^)	A/T—0.86 (0.83–0.89)	1: (0.00003/0.99): n/a	15 (3) *LOXL2*
				rs200262583 (3.22 × 10^−12^)	A/AGTCCTCCTTTTCTT— 0.90 (0.87–0.93)	0.88: (0.374/0.018): 0.019	49 (31)
11q13^a^ (11_3)	rs7931342 rs10896438 rs12793759	68811777–69494148	rs12275055 (4.7 × 10^−53^)	rs12275055 (6.1 × 10^−23^)	A/G—0.83 (0.79–0.87)	1: 0.19: n/a	5 (1)
				rs10792032 (3.5 × 10^−17^)	A/G—1.13 (1.10–1.16)	0.97: 0.90: 0.19	30 (4)
				rs36225067 (1.34 × 10^−8^)	A/C—0.80 (0.73–0.88)	0.75: 0.002: 0.002	16 (13)
12q24 (12_3)	rs1270884	114632506–115103229	rs1270884 (6.8 × 10^−9^)	rs1270884 (1.34 × 10^−8^)	A/G—1.08 (1.05–1.10)	1: 1: n/a	23 (0)
				rs61933115 (7.58 × 10^−6^)	A/G—1.09 (1.05–1.13)	0.52: 0.0008: 0.0008	1 (0)
14q24 (14_2)	rs7141529	68974508–69135467	rs7141529 (6.5 × 10^−11^)	rs7141529 (5.27 × 10^−12^)	T/C—0.92 (0.89–0.94)	1: 1: n/a	2 (1)
				rs2189517 (7.80 × 10^−6^)	A/G—1.06 (1.03–1.09)	0.96: 0.002: 0.002	8 (1)
				rs17105852 (7.16 × 10^−5^)	A/C—0.86 (0.79–0.94)	0.97: 0.001: 0.001	1 (1)
17q12^a^ (17_2)	rs11649743 rs4430796	35740855–36249855	rs11263763 (2.1 × 10^−66^)	rs11263763 (1.0 × 10^−62^)	A/G—1.25 (1.22–1.27)	0.97: (0.008/0.94): n/a	5 (3)
				rs718961 (5.35 × 10^−12^)	A/G—0.90 (0.87–0.93)	0.98: (0.76/0.005): 0.005	4 (4)
				rs2229295 (3.75 × 10^−7^)	T/G—1.10 (1.06–0.13)	0.96: (0/0): 0.0003	1 (1)
17q21 (17_3)	rs11650494	46845186–47936749	rs138263737 (7.0 × 10^−12^)	rs138263737 (5.7 × 10^−10^)	T/C—1.93 (1.72–2.14)	0.60: 0.002: n/a	1 (0)
				rs11655191 (1.82 × 10^−7^)	T/C—1.13 (1.09–1.18)	0.96: 0.76: 0.0004	70 (20) *ZNF652*
22q13 (22_2)	rs5759167	43000212–44000212	rs5759167 (1.8 × 10^−29^)	rs5759167 (6.5 × 10^−24^)	T/G—0.87 (0.85–0.90)	1: 1: n/a	2 (1)
				rs5751435 (4.55 × 10^−10^)	T/C—0.88 (0.84–0.92)	0.98: 0.02: 0.02	15 (6) *TTLL12*/*MCAT*

Following multiple stepwise logistic regression analyses, multiple independent association signals were identified in 16 regions. The *TERT* at Chr5p15 and Chr8q24 regions which are also known to harbor multiple independent PrCa susceptibility loci were not included in this analysis as they had previously been fine-mapped individually (6,7). Imputation quality and correlation (LD) between the novel lead SNPs and the original GWAS signal(s) are indicated, as are the correlations between the most strongly associated variant in this analysis and the additional independent hits within the region.

Best Candidate SNPs are variants correlated at *r*^2^ > 0.7 with the lead variant describing an association, and with odds of association ≥1/1000 relative to the lead variant for the region. eQTL data indicate statistically significant correlation between the new index SNP and gene expression in 145 prostate tumor samples from the TCGA dataset.

^a^These regions were densely genotyped on the iCOGS chip to fine-map PrCa associations known at the time of design.

For each of the 75 independent association signals identified across the 55 regions analyzed, we have selected a set of the most promising correlated candidate causal variants. Since a greater density of variants are interrogated during fine-mapping than through the ‘tag SNP’ approach used in the discovery phase of GWAS, the causal variants responsible for PrCa risk at each region would generally be expected to associate with PrCa at a similar level to the refined lead SNP, as well as exhibiting relatively high levels of linkage disequilibrium (LD). Consequently, we selected our ‘best candidate’ variants that most warrant further interrogation for functionality and PrCa causality for each association signal using an overlap between two criteria: likelihood ratio of ≥1/1000 relative to the refined lead SNP (‘1000 worse’ list = 6537 SNPs) and correlation with the lead SNP at LD *r*^2^ > 0.7 (LD list = 2202 SNPs). This best candidate list comprised 1623 SNPs across the 55 regions studied, with between 1 and 93 SNPs per association signal and a median of 13 candidate variants (Supplementary Material, Table S2). These best candidate SNPs were annotated for overlap with functional elements in PrCa cell lines. For this analysis, bio-features were annotated according to the methodology used in Hazelett *et al.*(14). Of our 1623 best candidate SNPs, 413 (25%) were either coding or within an annotated bio-feature, with enhancer elements accounting for the largest single class of element represented (Supplementary Material, Table S2). We also analyzed the best candidate SNP list for potential eQTLs using TCGA data available for prostate tissue and a much larger set of EuroBATS data for three different tissue types (skin, LCL and adipose). To aid in the interpretation of data from this study, we developed Locus Explorer, a Shiny R application which allows the interactive graphical illustration of all the regions we have fine-mapped and which can be accessed at https://github.com/oncogenetics/LocusExplorer (manuscript in preparation). In the Locus Explorer plots for this study, we have included LD structure, statistical association data, functional annotation, gene transcripts and eQTL data; however, this application is customizable as required.

**Figure 1.**
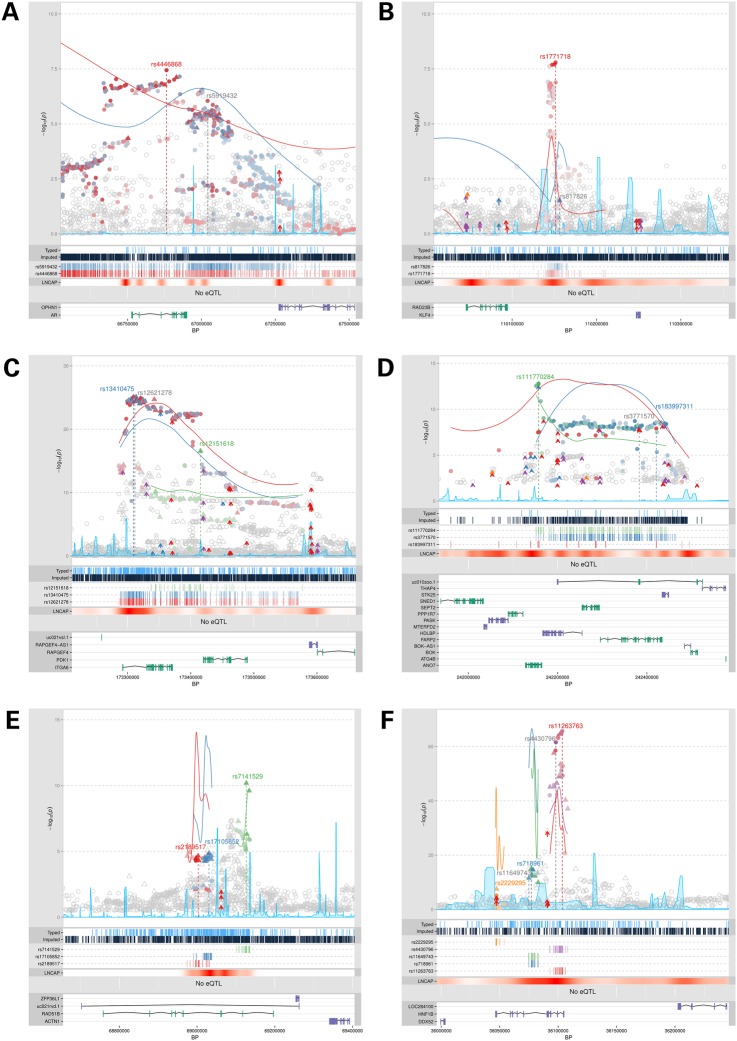
Locus explorer plots of two simple and four complex regions. (**A**) Region 23_3 at ChrXq12, (**B**) Region 9_1 at Chr9q31, (**C**) Region 2_6 at Chr2q31, (**D**) Region 2_8 at Chr2q37, (**E**) Region 14_2 at Chr14q24 and (**F**) Region 17_2 at Chr17q12. For regions containing multiple independent association signals, the separate lead SNPs are indicated and colored red, blue, green, orange and purple, respectively. Original GWAS tag SNPs that were replaced during fine-mapping are marked in gray on the plot. Clusters of correlated variants for each signal are distinguished using different colors in the plot and on the panel below, including for the original GWAS SNPs. Stronger shading indicates greater correlation with the lead SNP, with variants not correlated at *r^2^* ≥ 0.5 with any lead SNP uncolored. Directly genotyped variants are denoted as triangles and imputed variants as circles. Log_10_ *P*-values are shown on the *Y*-axis of the plot. Colored arrows within the plot mark SNPs that overlap with regulatory elements in ENCODE; red for 3′UTRs, blue for coding variants, purple for promoters and orange for miRNA sites. The position of genes within the region and the genomic coordinates of the plot are shown on the lower panel, with genes on the positive strand in green and the negative strand in purple. The LNCaP track shows the density of annotated bio-features within the LNCaP cell-line (data from ENCODE).

For 4 of the 39 simple regions, the originally reported SNP remained the most strongly associated variant after fine-mapping, whereas in the remaining 35 regions a new lead SNP was identified (Table 1). The novel lead variants were generally in strong (*r*^2^ > 0.7) or moderate LD (*r*^2^ = 0.3–0.7) with the original GWAS tag SNP; however, for five regions, the new lead SNP was only in weak LD with the original signal (*r*^2^ < 0.3). In 10 regions, the originally reported variant was excluded from our set of the best candidate SNPs to further investigate for possible causal functional effects. A good illustration of the refinement of the association signal within simple regions is at ChrXq12 where the original signal, rs5919432, was situated 71 kb 3′ of the androgen receptor (*AR*) gene. This has now been replaced by rs4446868 which is intronic to *AR* and in strong LD with a number of variants within the coding sequence, while the original tag SNP did not remain among our list of the best candidate functional variants (Table 1, Fig. 1A; Supplementary Material, Table S2). It may also be possible to further prioritize the 46 selected candidate SNPs at this region based on statistical and functional evidence; a cluster of five variants were more strongly associated than the remainder and these flank a known AR-binding site within intron 2 that has been reported to function as an enhancer in LNCaP (15).

Two of the simple regions had been reported previously as PrCa susceptibility variants only in East Asian populations (rs1983891 at Chr6p21 and rs817826 at Chr9q31); in this study, we have demonstrated for the first time that these loci are also associated with PrCa risk in individuals with European ethnicity. The lead SNP describing the association signal for Europeans at Chr6p21, rs6458228, is strongly correlated with the Japanese hit (*r*^2^ = 0.92); both are intronic in *FOXP4* and in LD with variants overlapping a number of bio-features. Our top European hit on Chr9q31 near *RAD23B*, rs1771718, is not correlated (*r*^2^ = 0.03) with the index SNP reported in the Chinese population and itself overlaps a DNase1 hypersensitivity site in the LNCaP PrCa cell line. Based on TCGA data, this signal is also an eQTL for *RAD23B* in normal prostate tissue but not tumor (Table 1 and Fig. 1B).

Of particular interest, within the 55 regions included in our final analysis, we identified 16 ‘complex regions’ that harbor more than one independent association signal after conditional analysis and contained a total of 36 separate risk signals (Table 2). Only five of these regions had previously been reported to contain independently associated SNPs (*CHMP2B* at Chr3p12 (8,16), *PDLIM5* at Chr4q22 (17), *SLC25A37/NKX3.1* at Chr8p21 (17), Chr11q13 (16,18) and *HNF1B* at Chr17q12 (19,20)). At both Chr3p12 and Chr8p21, the two originally reported association signals are within 50 kb of one another however situated adjacent to different genes. As this proximity fell comfortably within the flanking distance we defined in this study for each region they were nonetheless merged into a single region for this analysis. Multiple independent risk variants within the same gene had previously been identified within *PDLIM5* and *HNF1B*, while Chr11q13 is a gene desert previously reported to contain three independent hits. For all of these five regions that were known to be complex prior to our fine-mapping, in this study, we confirmed each of the previously reported independent associations, have refined the spectrum of variation that best describes these association signals, and we have identified a novel, third independent signal at *HNF1B*/Chr17q12. Furthermore, we have discovered additional novel signals within 11 regions previously known to contain only a single risk variant.

To illustrate the utility of fine-mapping in combination with functional annotation for the refinement of GWAS signals towards promising candidate causal variants, we present detailed findings for four representative complex regions in this manuscript. In the Chr2q31 region, we identified two independent signals (Table 2 and Fig. 1C). The most strongly associated SNP, rs13410475, is intronic in *ITGA6* and in complete LD with the original index SNP rs12621278 (*r*^2^ = 1). This signal represents a tight cluster of four potentially causative variants. The novel additional independent lead SNP, rs12151618, is weakly correlated with this signal (*r*^2^ < 0.07) and situated 5 kb upstream of *PDK1* (pyruvate dehydrogenase kinase isozyme 1) in the promoter region. *PDK1* is believed to be upregulated by *Myc* and *HIF-1* to facilitate cell survival and proliferation under hypoxia, and is reported to be commonly overexpressed in cancer cells (21,22).

Two independent signals were also identified at Chr2q37, where the previously reported hit rs3771570 was intronic in *FARP2* (Table 2 and Fig. 1D). In our analysis, the most strongly associated novel lead SNP, rs111770284, is not in LD with the original GWAS hit (*r*^2^ = 0.03). rs111770284 is intronic in *ANO7* (also known as *NGEP*, New Gene Expressed in Prostate), overlaps a DNase1 site in LNCaP and implicates a set of four correlated variants as putative functional candidates. *ANO7* is an androgen regulated gene and its expression appears to be prostate specific (23,24), with reduced expression associated with increased PrCa malignancy (25). The second independent signal, rs183997311, is intronic in *FARP2*. This variant is not in LD with either the original GWAS tag SNP or rs111770284. rs183997311 is relatively rare (MAF = 1%) and is correlated to two variants that overlap regulatory elements in LNCaP. It is also notable that the original hit rs3771570 was excluded from our list of best candidate causal SNPs after fine-mapping of this region.

At Chr14q24, the original signal rs7141529 remained the most significantly associated variant after stepwise logistic regression; however, two further additional independent signals described by rs2189517 and rs17105852 were identified (Table 2 and Fig. 1E). These novel variants are both situated within the same long intron of *RAD51B* but are not correlated with each other or with rs7141529. This region also harbors two independent risk signals for breast cancer, one of which is additionally associated with breast cancer in males; however, there is no correlation between these variants and any of the three PrCa risk SNPs (26,27) (Supplementary Material, Fig. S3).

The *HNF1B* locus on Chr17q12 contained two previously reported independent signals for PrCa. We identified more strongly associated lead SNPs to describe these signals, while the original GWAS SNPs were also excluded from the list of best candidate variants. The novel lead SNPs, rs11263763 in the first intron and rs718961 in the fourth intron, each overlap with multiple bio-features and therefore themselves represent good causal candidates, although both are also correlated to a modest number of other promising candidates (Table 2; Supplementary Material, Table S2). In addition to these refinements of the original signals, we have identified a previously unknown third independent association described by rs2229295, which lies within the 3′UTR of *HNF1B* and may itself represent a strong candidate causal variant worthy of further investigation (Fig. 1F).

**Figure 2.**
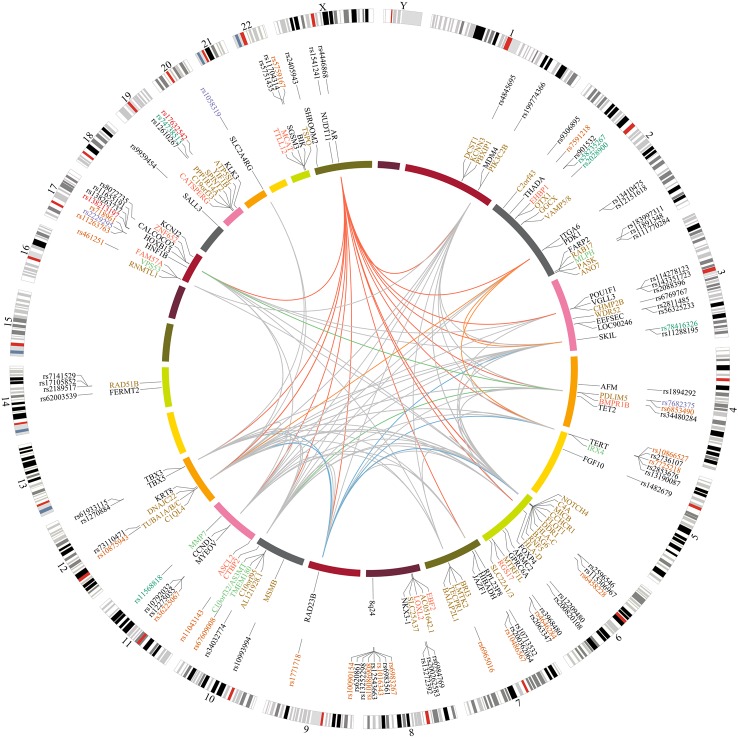
Circos plot overview of functional annotation and eQTL data for fine-mapped PrCa risk loci generated using Circos (http://circos.ca/, 62). The outer ring is a circular ideogram of the human genome annotated with chromosome number. The positions of the novel index SNPs for PrCa susceptibility identified through fine-mapping are indicated adjacent to this and are color coded for overlap with enhancer elements in LNCaP in orange, promoter regions in green, coding SNPs in red, variants within UTR regions in purple and variants with no annotated functionality in black. The inner ring denotes potential candidate genes for the refined PrCa regions. Genes for which an SNP in the best candidate list is a significant eQTL in prostate tissue in TCGA data are indicated in red, eQTLs in skin tissue from EuroBATS data are marked in brown, eQTLs for both prostate and skin in green and for regions with no significant eQTL in either tissue the closest flanking gene is indicated in black. Gene interaction networks between potential candidate genes are shown as links in the central portion of the plot. The genes annotated on the inner ring were used to construct a network using the BioGRID interaction database filtered to exclude ubiquitin and interactions with more than a single intervening gene between the candidate genes. Red links indicate an interaction network with the *AR* gene, other examples of interaction highlighted in color: blue—*RAD23B*, green*—BMPR1B*, orange—*PDK1* and all other interactions are marked in gray.

To further interrogate the variants in our best candidate list for potential functional effects, we examined TCGA data from 145 prostate tumor and 45 normal prostate tissue samples for differential expression of nearby genes associated with these SNPs. We observed significant associations with gene expression for 16 of our association signals (Fig. 2, Tables 1 and 2; Supplementary Material, Table S2). Several of these eQTLs have been reported previously (28,29) and most recently Li *et al.* described significant eQTLs at 31 of 69 PrCa GWAS regions they tested (45%), implementing a mapping strategy for 19 of these regions which selected candidate causal variants ranging from 1 to 33 SNPs (30,31). Here, we report prostate tissue eQTLs at ∼20% of the loci we analyzed; however, this is likely to be an underestimate due to the relatively small set of prostate samples currently available in TCGA (30). We subsequently examined EuroBATS (32,33) eQTL data for a much larger set of samples from lymphoblastoid cell lines (814 samples), skin (716 samples) and adipose (766 samples) tissues in order to investigate whether any of the eQTLs identified in the TCGA prostate tumor tissue dataset act ubiquitously and also whether additional signals are present within these tissues that might also be detectable in a larger set of prostate samples. Only one region (Chr10q24 for *C10orf32*) showed evidence for ubiquitous eQTL association in all four tissue types interrogated. There were nine signals across seven regions which had at least one concordant eQTL between the TCGA PRAD tumor and any tissue type in EuroBATS. Of the eQTL signals which are confirmed in both TCGA and at least one EuroBATS tissue, five have been described before using the TCGA dataset (*AS3MT*, *VPS53*, *MLPH*, *IRX4* and *RGS17*) (30). We therefore consider the four novel signals identified here (*C10orf32*, *TMEM180*, *MMP7* and *TTLL12*) as robust candidates for functional follow-up, especially due to the large sample set used in the EuroBATS project (Supplementary Material, Table S2). *TTLL12* is a particularly interesting candidate susceptibility gene as its expression has been shown to increase during cancer progression and metastasis (34). In addition to these eQTLs replicated between the two separate datasets, we also report a strong eQTL for *ZNF652* within the TCGA prostate tissue data solely. We have also detected eQTLs for *RAD51B* within the EuroBATS data only and for *RAD23B* in normal prostate TCGA data, but not in prostate tumor. These potentially interesting associations may therefore warrant further follow-up within a larger prostate tissue set. In the central portion of our circos plot (Fig. 2), we have illustrated potentially interesting interaction networks between the candidate genes identified through this fine-mapping study. This highlights in particular that a large number of these plausible candidate genes are regulated by the AR, as shown in red.

To evaluate whether the refinement of previously identified PrCa loci that we have achieved in this study reflects a greater enrichment towards variants with annotated functionality, we assessed our list of 1623 best candidate SNPs for overlap with bio-features and compared this against the full set of imputed SNPs within the 55 regions that were significant in this analysis (243 627 SNPs). Using the hypergeometric test, we observed a significant enrichment in overlap with bio-features for the variants in our best candidate SNP list (*P* = 2.01 × 10^−20^, Supplementary Material, Fig. S4). This enrichment was even stronger when only enhancer elements were included in the analysis (*P* = 1.67 × 10^−27^).

In order to estimate the extent to which fine-mapping of these known PrCa susceptibility regions could improve understanding of the genetic factors that influence PrCa risk, we compared the FRR of PrCa explained by the original GWAS tag SNPs with the FRR explained by the novel lead SNPs that we have identified in this study. We used estimated variant effect sizes and allele frequencies from the samples in the iCOGS study to calculate both estimates to avoid the potential for inflation from the UK GWAS study. We accounted for the LD between variants in the complex regions containing more than one independent SNP to avoid overestimating FRR. The estimated FRR explained when substituting for our refined lead SNPs and introducing the newly identified independently associated variants from this study was 38.9%, compared with 30% for the originally reported GWAS tag SNPs; an improvement of 8.9% overall, and nearly a third greater than had been previously attributed to these known PrCa susceptibility signals.

## Discussion

In this study, we used imputation of existing genotype data to fine-map 64 PrCa GWAS regions in European ancestry populations comprising 25 723 PrCa cases and 26 274 controls from three studies (iCOGS and UK GWAS Stages 1 and 2). Twenty-three of these regions were fine-mapped at very high resolution on the iCOGS chip. Nine previously reported GWAS signals were not replicated at genome-wide significance in this study due to either decreased power in comparison with the original studies or having only previously been associated with PrCa susceptibility in a non-European population.

In 39 of the remaining 55 regions, we found evidence for a single PrCa association signal only. The original GWAS tag SNP remained the most significant association for just 4 of these, while at 35 regions, we identified a more significantly associated replacement lead SNP. Importantly, we also identified 16 complex regions containing multiple variants independently associated with PrCa. Only five of these had previously been identified as containing multiple independent variants (Chr3p12, Chr4q22, Chr8p21, Chr11q13 and Chr17q12); however, our analysis helped to further refine these five regions and identified one additional previously unknown hit at Chr17q12 within the promoter region of *HNF1B*. For the remaining 11 complex regions, this study provides the first evidence for the presence of multiple independent PrCa susceptibility variants in close genomic proximity to one another. An alternative explanation for the observation of multiple apparently independent association signals would be that both could in fact be moderately correlated with a single untyped causal variant, despite exhibiting limited LD between themselves (35,36). We believe that this hypothesis is unlikely to underpin a substantial proportion of the multiple signals we have observed in this study, since 11 of the 16 identified complex regions had been densely genotyped on the iCOGS array, enabling very thorough imputation of additional untyped variants within the region. However, this phenomenon cannot be completely excluded without deep re-sequencing of each of these loci in a large sample panel, which would facilitate the evaluation of all correlated variation within the region and subsequently the identification of the precise causal variants. Our functional annotation of the statistically most promising correlated candidate causal variants also provides provisional evidence to implicate a contribution by several new potential candidate genes in PrCa risk.

We have annotated our set of statistically significant SNPs in order to prioritize the most likely candidate causal variants within each region. We firstly excluded all SNPs that were associated with PrCa risk at a conservative threshold of ≤1/1000 compared with the association likelihood of the novel lead SNP for each signal. This generated a list of 6537 variants across the 55 significantly associated regions in our final analysis. To further prioritize within this list, we trimmed based on LD structure and selected only those variants that were strongly correlated (LD *r*^2^ > 0.7) with the lead SNP based on 1 KG EU data. The intersection of these two selection criteria generates a list of 1623 variants, which we would expect to retain and be enriched for the causal functional variants. However, we cannot exclude the possibility that in some instances the causal variant(s) may be in lower LD with the novel index variants, particularly in the regions that were less densely genotyped. In addition, our imputed association data may be underpowered to detect any instances where rare causal variants give rise to the association signal through a ‘synthetic association’ (37). The incorporation of functional annotation in addition to statistical data and LD criteria may also help to further prioritize our list of the best candidate SNPs for PrCa risk causality towards those with the strongest evidence for biological effect, to facilitate potential laboratory follow-up. For 62 of the 75 independent association signals we detected, one or more of the best candidate variants in our list overlaps with bio-features in PrCa cell lines (413 of the 1623 variants were annotated for functionality in total, median of 4 per signal). We furthermore observed eQTLs for differential gene expression in TCGA prostate tumor or normal tissue for one or more variants in our best candidate list for 16 of these 75 independent association signals, with additional eQTLs at other regions also observed within a larger set of EuroBATS data for three additional tissues.

An important aspect of this mapping study is the improvement of the estimated FRR of PrCa explained by the refined and newly identified independently associated variants. This is now substantially higher, at ∼39% compared with 30% estimated for the original GWAS tag SNPs (12). Fine-mapping of these 55 known regions has therefore improved our understanding of the genetic basis of PrCa and incorporating these novel variants into future risk models should enhance the capability to predict individuals at greater risk. It is also interesting to note that of the 23 regions analyzed in this study that were known at the time of design of the iCOGS array and therefore fine-mapped through a more dense set of directly genotyped markers; these represent 11 of the 16 complex regions and only 12 of the 39 simple regions. This might suggest that the presence of multiple independent PrCa susceptibility variants within previously identified GWAS regions could be even more widespread than we have been able to identify in this fine-mapping study. Consequently, additional susceptibility signals could yet reside within the regions that remain to be interrogated through very dense marker resolution in a sufficient sample size; an experiment which is currently being undertaken by the OncoArray Consortium (38).

A concurrent PrCa fine-mapping study by Han *et al*. (*Hum. Mol. Genet*., submitted) has examined 69 risk regions among a multi-ethnic sample panel comprising European (8600 cases and 6946 controls), African (5327 cases and 5136 controls), Asian (2563 cases and 4391 controls) and Latino (1034 cases and 1046 controls) ethnicities. After performing a meta-analysis for marginal tests across multiple populations 12 regions were not significant, while a single novel, more significantly associated lead SNP was identified at 32 of the 57 significant regions (56.1%). In comparison, across the 55 regions that achieved genome-wide significance in our study, the original GWAS tag SNP was replaced with a more significantly associated SNP at 47 (85.4%). For the 46 significant regions that overlapped between these two studies’ final datasets, 32 of the refined SNPs identified by Han *et al.* were among our list of best candidate SNPs (69.5%). Within these overlapping regions, we identified 12 novel independent signals, 9 of which were nominally significant in the multi-ethnic fine-mapping but none were included in their top order or putatively functional SNPs. Comparing final putative functional candidates for the 46 overlapping regions, 29 (63.0%) of these regions have at least one overlapping functional candidate SNP. More notably, 11 of these 46 regions (23.9%) have a single overlapping functional candidate SNP, which should therefore be assigned high priority as potential causal candidates for future experimental follow-up. Overall, the comparisons between these two approaches highlight the increased power provided by the larger sample set available within our study; in particular in respect to the identification of multiple independent association signals within already known regions, but also demonstrate that the ability to incorporate multiple ethnic populations may further improve the efficiency of fine-mapping. This suggests that large meta-analysis based fine-mapping studies comprising individuals of diverse ancestries may represent the most robust strategy for imputation-based fine-mapping where such data are available.

In conclusion, we have demonstrated the importance of genotyping and imputation-based fine-mapping through the discovery of 12 additional independent PrCa associations within known GWAS regions and by refining the vast majority of the previously reported signals. Before fine-mapping efforts were employed, potential causal SNPs at each susceptibility locus were selected primarily on the basis of high correlation to the reported GWAS tag SNP (14) or overlap with functional elements; this strategy can however either introduce substantial noise or a greater likelihood of excluding true causal variants, due to the lack of statistical evidence available when retaining or excluding non-genotyped variants. Through coupling functional annotation and LD information to our imputed association dataset, we believe we have been able to enrich for the most likely candidate causal PrCa susceptibility variants at each association signal while substantially reducing noise within the list. Indeed, this approach selected only a modest number of SNPs per locus, yet these showed evidence for modulating differential gene expression and greater overlap with bio-features in prostate datasets. While complete re-sequencing of GWAS regions in large sample sets would ultimately be desirable, our approach undoubtedly represents the most time and cost-effective method available at the present time to interrogate and prioritize the most plausible causal variants underpinning GWAS studies. We have demonstrated that many of the GWAS regions identified to date for PrCa harbored additional, previously hidden independent association signals, with perhaps more yet to be discovered. This observation may have important implications towards the deconvolution of the functional mechanisms underlying these signals, and in particular could help to facilitate improved risk prediction, since these additional independent association signals could account for a significant proportion of the missing heritability of PrCa and other complex diseases (39).

## Materials and Methods

### Samples

Analyses were based on data from the iCOGS array, a custom array comprising ∼200 000 SNPs designed to study susceptibility to prostate (PRACTICAL Consortium), breast and ovarian cancers (12), together with data from a UK GWAS study (Stage 1) and subsequent custom array designed as a replication stage of the Stage 1 GWAS that was genotyped in studies from the UK and Australia (Stage 2). The analyses presented here were restricted to 51 997 men (25 723 cases and 26 274 controls) of European ancestry, samples with other ethnicity in the iCOGS study were small in number and were included in a separate study addressing mapping using multi-ethnic sample sets (Han *et al*., *Hum. Mol. Genet*., submitted). See Eeles *et al*. (12) for details of the quality control (QC) procedures. After QC, data were available for 11 338 samples (5504 cases and 5834 controls) from the GWAS, and 40 659 samples (20 219 cases and 20 440 controls) from 32 studies in PRACTICAL genotyped using the iCOGS array. The majority of studies were population- or hospital-based case–control studies, or nested case–control studies, but some studies selected samples by age or oversampled for cases with a family history; in the latter instance only one case per family was genotyped (Supplementary Material, Table S5). Only the 40 659 iCOGS samples were used for the FRR estimation to avoid inflation from the Stage 1 GWAS.

### Genotyping

UK GWAS Stages 1 and 2 were genotyped using the Illumina Infinium HumanHap550 and a custom Illumina iSELECT array, respectively (17,40).

*iCOGS* genotyping was conducted using a custom Illumina Infinium array (iCOGS) in seven centers, of which five were utilized for PRACTICAL. Genotypes were called using Illumina's proprietary GenCall algorithm. In addition to SNPs selected for replication of GWAS, the iCOGS array included dense sets of SNPs surrounding susceptibility variants known at the time of design. For PrCa, we included 23 such regions (ccge.medschl.cam.ac.uk/research/consortia/icogs/). To select markers for comprehensive interrogation of these densely genotyped regions, we identified all known SNPs from the March 2010 release of the 1000 Genomes Project with MAF > 0.02 in Europeans and selected all SNPs that were correlated with the published GWAS SNPs (at *r*^2^ > 0.1); together with a set of SNPs designed to tag all remaining variants at *r*^2^ > 0.9. Approximately 14 000 SNPs were successfully designed across these regions.

### Statistical methods

The primary genotype data were used to impute genotypes for ∼17 M SNPs/indels using the 1000 Genomes Project (March 2012 release) as a reference panel and IMPUTE V2 (41). Imputation was carried out using pre-phasing with 50 iterations and all known PrCa regions were imputed in an ∼5 Mb block size. Per-allele odds ratios and standard errors were estimated for each SNP by logistic regression. Analysis was stratified by study and, for both the GWAS and iCOGS, included eight principal components as covariates. We included imputed data for SNPs with quality information scores >0.3. Analyses were performed using SNPTEST (42). Results from the iCOGS and GWAS were then combined in a fixed effect meta-analysis using METAL (43).

### Comparison of number of associated loci among cancer-specific regions

To establish a suitable significance threshold, we examined 59 known breast cancer regions with no known association with PrCa prior to this analysis. Regions were defined as a ±500 kb boundary around the published breast cancer GWAS SNPs. We interrogated our meta-analysis results (iCOGS and Stages 1 and 2 UK GWAS) in order to establish the likelihood that SNPs within these breast cancer regions would be associated with PrCa by chance at *P* ≤ 10^−5^ level. Performing this comparison revealed that an association *P* = 10^−5^ was sufficiently strict for the avoidance of false-positive results in the discovery of secondary signals within a region after adjusting for the top signal variant.

### Identification of lead SNPs within each region

To identify independent association signals within a region, we selected all significant SNPs with *P* ≤ 10^−5^ from the fine-mapping dataset (iCOGS, Stages 1 and 2 GWAS) and performed a stepwise logistic regression on this set of SNPs for each region. For regions in which the initial analysis indicated more than one independent SNP, a second round stepwise logistic regression was performed to confirm the presence of additional independent SNPs, after adjusting for the best signal in the region. SNPs with *P* ≤ 10^−5^ from the adjusted results were included in this analysis, alongside the top hit. For regions found to contain multiple independently associated SNPs, haplotype-specific odds ratios were estimated based on the independent SNPs identified through the stepwise logistic regression analysis, using the Haplo.Stats package (http://cran.r-project.org/web/packages/haplo.stats/index.html) (44). SNPs with a likelihood ratio of ≥1/1000 relative to the most significant SNP describing each signal were selected as potential candidate causal variants (45). This list was narrowed down further by filtering variants at LD *r*^2^ < 0.7 with the lead SNPs to curate a list of best candidate causal SNPs for each signal. For regions with multiple independent SNPs, the lists for the subsequent signals were generated by determining the likelihood ratio relative to the top SNP in that region, adjusted for other signals. We excluded duplicates and used a cut-off *P* = 6.7 × 10^−5^ (*P*-value for odds of >1/1000 relative to the genome-wide significance level of 5 × 10^−8^) to trim the list where there was a second signal that did not reach the genome-wide significance level (5 × 10^−8^).

### Contribution to familial risk

The contribution of SNPs to the familial risk of PrCa, under a multiplicative model, was computed using the formula

$$\frac{\sum_{k}\log\lambda_{k}}{\log\lambda_{0}},$$

where $\lambda_{0}$ is the observed familial risk to first-degree relatives of PrCa cases, assumed to be 2 (46,47), and $\lambda_{k}$ is the FRR due to locus *k*. For a single SNP, $\lambda_{k}$ is given by

$$\lambda_{k}=\frac{\;p_{k}r_{k}^{2}+q_{k}}{{\left(\;p_{k}r_{k}+q_{k}\right)}^{2}}$$

where $\;p_{k}$ is the frequency of the risk allele for locus *k*, $q_{k}=1-\;p_{k}$ and $r_{k}$ is the estimated per-allele odds ratio, estimated from the logistic regression model for that SNP. For regions with more than one SNP in LD, we used the extended formula:

$$\lambda_{k}=\frac{\sum_{j}\;p_{kj}r_{kj}^{2}}{{\left(\sum {\;}_{j}\;p_{kj}r_{kj}\right)}^{2}}$$

where $\;p_{kj}$ is the frequency of haplotype *j* for the multi-locus region *k* and $r_{kj}$ is the corresponding risk estimate. The haplotype frequencies were estimated using Haplo.Stats, while the haplotype-specific risk estimates were based on the regression analyses, thus preserving the assumption of a log-additive effect of all SNPs.

### Locus explorer

We developed Locus Explorer, a Shiny web application for R, to generate the locus plots shown in this manuscript. Locus Explorer can be run locally using R studio with the necessary packages installed. The code is available on GitHub. The application is interactive and enables selecting and zooming on the locus, selecting annotation tracks and downloading the resulting plots and the data used for plotting. Data for plotting are stored in SQLite database which is accessed using R scripts. Plots are displayed mainly using RStudio, sqldf, ggplot and ggbio (48–51). At the time of submission, the application is currently in Beta stage for external users, with most functionality in place and performance issues resolved. We are continuously working on additional features and for future versions the main focus will be speed of the plotting process. More information is available at GitHub (https://github.com/oncogenetics/LocusExplorer).

### Functional annotation

We used a number of publicly available prostate epithelia and PrCa ENCODE datasets of chromatin features to identify putative regulatory regions at each risk locus (14,52). The integration of chromatin bio-feature annotations with SNP positions was performed using FunciSNP (53). These datasets included LNCaP and RWPEI DNase I HS sites (GSE32970) ENCODE; PrEC DNase I HS sites (GSE29692) ENCODE; LNCaP CTCF ChIP-seq peaks (GSE33213) ENCODE; LNCaP H3K27ac and TCF7L2 (GSE51621) (14), H3K4me3 and H3K4me1 histone modification ChIP-seq peaks (GSE27823) (54); FoxA1 ChIP-seq peaks (GSE28264) (55); AR ChIP-seq peaks (56) and AR binding sites (GSE28219) (57); NKX3-1 ChIP-seq peaks (GSE28264) (55). We also used the highly conserved set of predicted targets of microRNA targeting (miRcode 11 June 2012 release) (58). To determine whether any of the putative functional SNPs potentially affect the binding of known transcription factors, position-specific frequency matrices were employed from Factorbook (14,59).

### cis-eQTL analysis

#### A/TCGA PRAD samples

The genotypes of the variants in the best candidate SNP list in 145 prostate tumor samples and 45 normal tissue samples were downloaded from TCGA database (Feb 2013). All samples were verified as Caucasian descendants. If a variant was not represented in the TCGA data, the genotypes were imputed using IMPUTE2. A cis-eQTL analysis was performed for these variants and any transcript within a 1 Mb interval (500 kb on either side). The abundance of the transcripts is adjusted for somatic copy number changes and CpG methylation changes using methods described previously. The nominal *P*-values obtained for each risk variant were corrected for the number of transcripts in the interval using Benjamini–Hochberg method. Significant associations were defined as a false discovery rate < 0.05.

#### B/EuroBATS samples

The sample set includes LCLs (*N* = 814), skin (*N* = 716) and adipose tissue (*N* = 766) derived simultaneously from a subset of well-phenotyped healthy female twins (32,33). eQTL discovery is described in detail elsewhere (32). In short, (i) we kept the residuals of a mixed model that removed the effects of the family structure using the implementation in GenAbel R package. (ii) We performed a linear regression of those residuals on the SNPs in a 1 Mb window around the transcription start site for each gene, using MatrixeQTL R package (60). We assessed statistical significance through 2000 permutations. Prior to the analysis, we removed the effects of technical covariates using the factor analysis strategy implemented in PEER (61) and transformed the data using a rank normal transformation.

## Supplementary Material

Supplementary Material is available at *HMG* online.

## Funding

This work was supported by European Commission's Seventh Framework Programme grant agreement No. 223175 (HEALTH-F2-2009-223175), Cancer Research UK Grants C5047/A7357, C1287/A10118, C5047/A3354, C5047/A10692, C16913/A6135 and The National Institute of Health (NIH) Cancer Post-Cancer GWAS initiative grant: No. 1 U19 CA 148537-01 (the GAME-ON initiative). Funding to pay the Open Access publication charges for this article was provided by the Institute of Cancer Research.

## Supplementary Material

Supplementary Data
