# Supplementary material for: Multiple novel prostate cancer susceptibility signals identified by fine-mapping of known risk loci among Europeans
Source: Hum Mol Genet. 2015 May 29;24(19):5589–602. doi: 10.1093/hmg/ddv203 (PMC4572072; doi:10.1093/hmg/ddv203)
Supplement: Supplementary Data [file supp_24_19_5589__index.html]

Multiple novel prostate cancer susceptibility signals identified by fine-mapping of known risk loci among Europeans — Multiple novel prostate cancer susceptibility signals identified by fine-mapping of known risk loci among Europeans — Supplementary Data 

# Multiple novel prostate cancer susceptibility signals identified by fine-mapping of known risk loci among Europeans

## Supplementary Data

Supplementary Data

- Supplementary File 1 - docx file
- Supplementary File 2 - xlsx file
- Supplementary File 3 - tiff file
- Supplementary File 4 - tiff file
- Supplementary File 5 - xlsx file
- Supplementary File 6 - docx file
