# Supplementary material for: Multiple novel prostate cancer susceptibility signals identified by fine-mapping of known risk loci among Europeans
Source: Hum Mol Genet. 2015 May 29;24(19):5589–602. doi: 10.1093/hmg/ddv203 (PMC4572072; doi:10.1093/hmg/ddv203)
Supplement: Supplementary Data [file supp_ddv203_ddv203supp_data1.docx]

**S1 Table – List of PrCa regions that did not achieve genome wide significance in this study**

For nine regions, no variant reached the threshold for genome wide significance in our European fine-mapping dataset (5.0×10^-8^). Four regions (10_3, 11_2, 13_1, and 19_4) are hits previously reported only for East Asian populations. Four regions (2_1, 3_4, 5_4, 20_1) were reported in previous studies by the PRACTICAL Consortium where larger sample sizes and therefore greater power to detect associations were available. Region 9_2 was published in a study where significance was achieved only for aggressive disease.

| **Chr (identifier)** | **Known Previous hit/ (P-value from a larger meta-analysis)** | **No.** | **Best Signal in fin×10-mapping (p-value) / (p-value form a larger meta-analysis)** | ***r^2^*** | **Original Publication** |
| --- | --- | --- | --- | --- | --- |
| 2p25 (2_1) | rs11902236 / (3.3×10^-7^) | 57 | rs7601896 (1.0×10^-6^)/ (1.6×10^-7^)) | <0.8 | Eeles et al. 2013 |
| 3p21(3_4) | rs6763931 /(9.96×10^-7^) | 23 | rs4683606 (1.1×10^-5^)/ (5.5×10^-7^) | 0.98 | Kote-Jarai et al. 2011 |
| 5q35(5_4) | rs6869841 / (1.1×10^-6^) | 11 | rs10067247 (2.0×10^-6^)/ (8.3×10^-7^) | 0.995 | Eeles et al. 2013 |
| 9q33(9_2) | rs1571801/ (7.0×10^-5^) | 2 | chr9:124654402:I (5.1×10^-4^) /(9.6×10^-3^) | <0.8 | Duggan et al. 2007 |
| 10 (10_3) | rs2252004 | 22 | chr10:122783141:I (1.8×10^-5^)/ (3.0×10^-6^) | <0.8 | Akamatsu et al. 2012 |
| 11 (11_2) | rs1938781 | 2 | rs148483367 (7.7×10^-4^)/ (7.7×10^-4^) | 0.006 | Akamatsu et al. 2012 |
| 13 (13_1) | rs9600079 | 13 | rs1571556 (3.2×10^-5^)/ (9.9×10^-5^) | 0 | Takata et al. 2010 |
| 19(19_4) | rs103294 | 2 | rs597744 (1.1×10^-5^) / (1.1×10^-5^) | 0.008 | Xu J et al. 2012 |
| 20q13 (20_1) | rs2427345 / (6.6×10^-8^) | 30 | rs2427345 (1.2×10^-7^)/ (6.6×10^-8^) | 1 | Eeles et al. 2013 |
