# Supplementary figures and images for: Multiple novel prostate cancer susceptibility signals identified by fine-mapping of known risk loci among Europeans
Source: Hum Mol Genet. 2015 May 29;24(19):5589–602. doi: 10.1093/hmg/ddv203 (PMC4572072; doi:10.1093/hmg/ddv203)

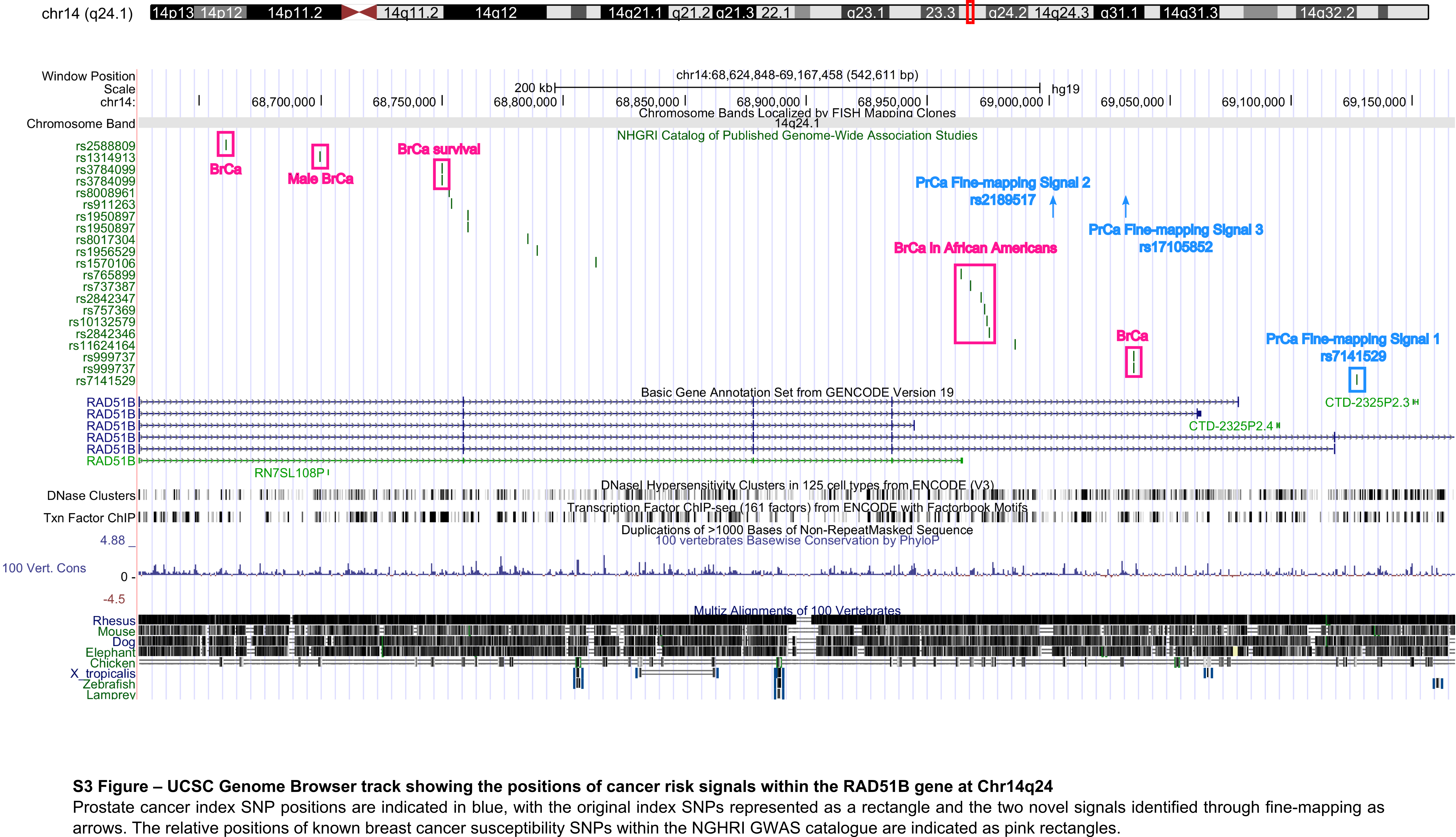

Supplement: Supplementary Data [file supp_ddv203_ddv203supp_data3.tif]

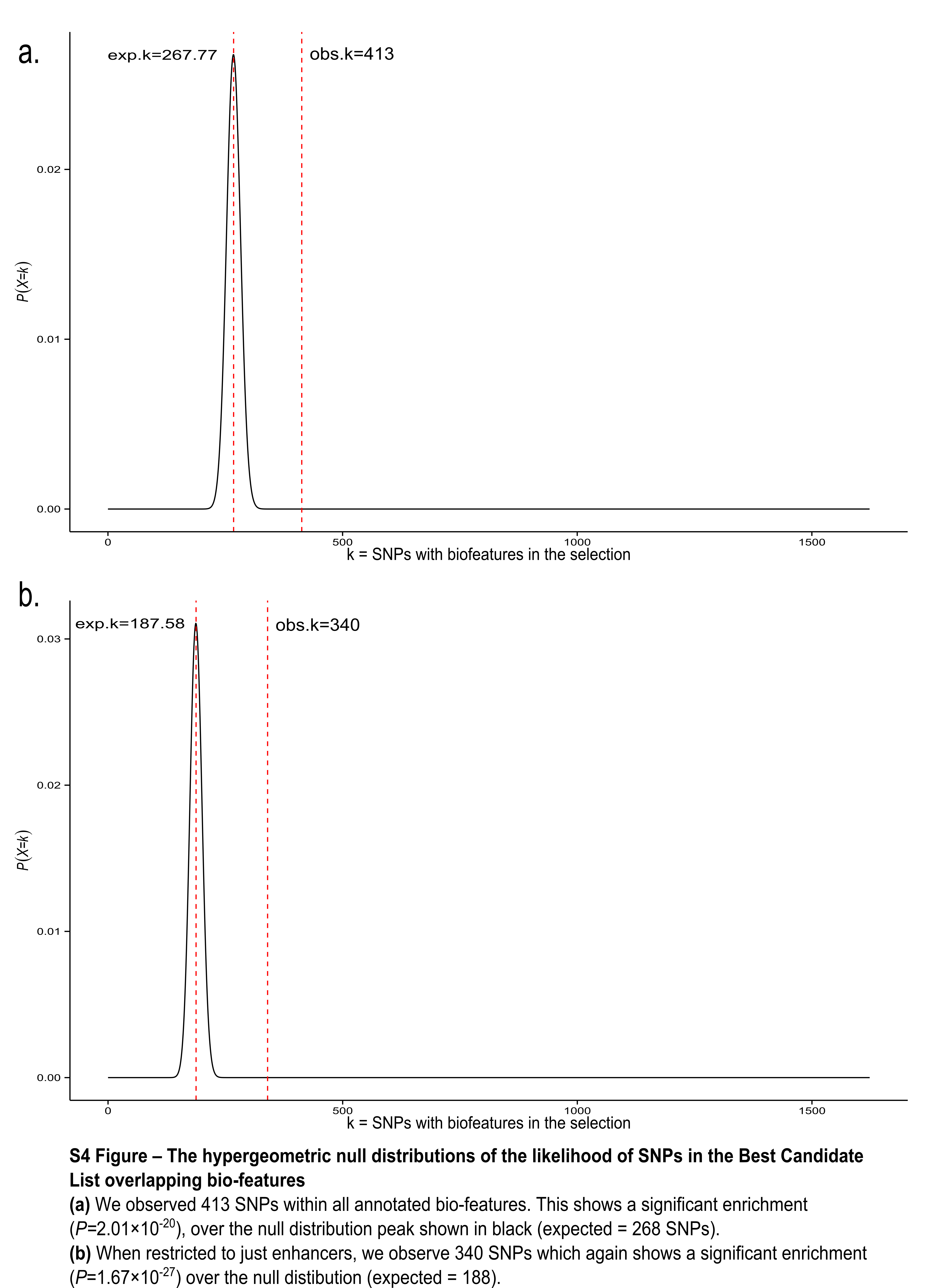

Supplement: Supplementary Data [file supp_ddv203_ddv203supp_data4.tif]
