# Supplementary material for: Multiple novel prostate cancer susceptibility signals identified by fine-mapping of known risk loci among Europeans
Source: Hum Mol Genet. 2015 May 29;24(19):5589–602. doi: 10.1093/hmg/ddv203 (PMC4572072; doi:10.1093/hmg/ddv203)
Supplement: Supplementary Data [file supp_ddv203_ddv203supp_data6.docx]

| **S6 Note –** **List of members of consortia that have contributed to this work and details of additional funding, URLs**  **List of members of consortia that have contributed to this work**  **The PRACTICAL CONSORTIUM (in addition to those named in the author list)**  Additional members from the consortium are: Margaret Cook^1^; Angela Morgan^2^, Artitaya Lophatananon^3,4^, Cyril Fisher^2^, Emma J. Sawyer^2^, Malgorzata Tymrakiewicz^2^, Naomi Livni^2^, Rosemary Wilkinson^2^, Sara Jugurnauth-Little^2^, Steve Hazel^2^, Gianluca Severi^5,6^, John Pedersen^5^, Melissa C. Southey^7^, Liesel M. Fitzgerlad^5^, John L. Hopper^8^; Ami Karlsson^9^, Carin Cavalli-Bjoerkman^9^, Jan-Erik Johansson^9^, Jan Adolfson^9^, Markus Aly^9,15^, Michael Broms^9^, Paer Stattin^9^; Brian E. Henderson^10^, Fredrick Schumacher^10^; Anssi Auvinen^11^, Kimmo Taari^12^, Liisa Maeaettaenen^13^, Paula Kujala^14^, Teemu Murtola^16,17^, Teuvo LJ Tammela^17^, Tiina Wahlfors^18^; Maren Weischer^19^, Andreas Roder^20^, Peter Iversen^20^, Peter Klarskov^21^, Sune F. Nielsen^19,22^; Tim J. Key^23^, Hans Wallinder^24^, Sven Gustafsson^24^; Jenny L. Donovan^25^, Freddie Hamdy^26^, Angela Cox^27^, Anne George^28^, Athene Lane^28^, Gemma Marsden^26^, Michael Davis^25^, Paul Brown^25^; Paul Pharoah^29^; Sarah Holt^30^; Lisa B. Signorello^31,37^, Wei Zheng^32^; Shannon K. McDonnell^33^, Daniel J. Schaid^33^, Liang Wang^33^, Lori Tillmans^33^, Shaun Riska^33^; Antje Rinckleb^34^, Kathleen Herkommer^35^, Manuel Luedeke^34^, Walther Vogel^36^; Dominika Wokozorczyk^38^, Jan Lubinski^38^, Wojciech Kluzniak^38^;, Christa Stegmaier^41^,; Babu Zachariah^43^, Hui-Yi Lim^44^, Hyun Park^43^, James Haley^43^, Julio Pow-Sang^43^, Maria Rincon^43^, Selina Radlein^43^, Thomas Sellers^43^; Aleksandrina Vlahova^45^, Atanaska Mitkova^46^, Chavdar Slavov^47^, Darina Kachakova^46^, Elenko Popov^47^, Svetlana Christova^45^, Tihomir Dikov^45^, Vanio Mitev^46^; Allison Eckert^48^, Amanda Spurdle^49^, Angus Collins^48^, APCB BioResource^48^, Glenn Wood^48^, Greg Malone^48^, Judith A. Clements^48^, Kimberly Alexander^48^, Kris Kerr^48^, Mary-Anne Kedda^48^, Megan Turner^48^, Pamela Saunders^48^, Peter Heathcote^48^, Srilakshmi Srinivasan^48^, Tracy Omara^48^, Trina Yeadon^48^; Joana Santos^50^, Joao Barros-Silva^50^, Paula Paulo^50^, Pedro Pinto^50^, Rui Henrique^50^, Sofia Maia^50^; Agnieszka Michael^51^, Andrzej Kierzek^51^, Huihai Wu^51^.  ^1^Centre for Cancer Genetic Epidemiology, Department of Public Health and Primary Care, University of Cambridge, Strangeways Laboratory, Worts Causeway, Cambridge CB1 8RN, UK, ^2^The Institute of Cancer Research, Sutton, UK, ^3^Institute of Population Health, University of Manchester, Manchester, UK, ^4^ Warwick Medical School, University of Warwick, Coventry, UK, ^5^Cancer Epidemiology Centre, The Cancer Council Victoria, 1 Rathdowne Street, Carlton, Victoria, Australia, ^6^Centre for Molecular, Environmental, Genetic and Analytic Epidemiology, The University of Melbourne, Victoria, Australia, ^7^Genetic Epidemiology Laboratory, Department of Pathology, The University of Melbourne, Grattan Street, Parkville, Victoria 3010, Australia, ^8^Centre for Epidemiology and Biostatistics, Melbourne School of Population and Global Health, The University of Melbourne, Victoria, Australia, ^9^Department of Medical Epidemiology and Biostatistics, Karolinska Institute, Stockholm, Sweden, ^10^Department of Preventive Medicine, Keck School of Medicine, University of Southern California/Norris Comprehensive Cancer Center, Los Angeles, California, USA, ^11^Department of Epidemiology, School of Health Sciences, University of Tampere, Tampere, Finland, ^12^Department of Urology, Helsinki University Central Hospital and University of Helsinki, Helsinki, Finland, ^13^Finnish Cancer Registry, Helsinki, Finland, ^14^Fimlab Laboratories, Tampere University Hospital, Tampere, Finland, ^15^Department of Clinical Sciences at Danderyds Hospital, Stockholm, Sweden, ^16^School of Medicine, University of Tampere, Tampere, Finland, ^17^Department of Urology, Tampere University Hospital, Tampere, Finland, ^18^BioMediTech, University of Tampere and FimLab Laboratories, Tampere, Finland, ^19^Department of Clinical Biochemistry, Herlev Hospital, Copenhagen University Hospital, Herlev Ringvej 75, DK-230 Herlev, Denmark, ^20^Copenhagen Prostate Cancer Center, Department of Urology, Rigshospitalet, Copenhagen University Hospital, Tagensvej 20, 7521, DK-2200 Copenhagen, Denmark, ^21^Department of Urology, Herlev Hospital, Copenhagen University Hospital, Herlev Ringvej 75, DK-230 Herlev, ^22^Denmark, Faculty of Health and Medical Sciences, University of Copenhagen, ^23^Cancer Epidemiology Unit, Nuffield Department of Clinical Medicine, University of Oxford, Oxford, UK, ^24^Department of Epidemiology and Biostatistics, School of Public Health, Imperial College, London, UK, ^25^ School of Social and Community Medicine, University of Bristol, Canynge Hall, 39 Whatley Road, Bristol, BS8 2PS, UK, ^26^Nuffield Department of Surgical Sciences, University of Oxford, Oxford, UK, Faculty of Medical Science, University of Oxford, John Radcliffe Hospital, Oxford, UK, ^27^CR-UK/YCR Sheffield Cancer Research Centre, University of Sheffield, Sheffield, UK, ^28^University of Cambridge, Department of Oncology, Box 279, Addenbrooke's Hospital, Hills Road Cambridge CB2 0QQ, UK, ^29^Centre for Cancer Genetic Epidemiology, Department of Oncology, University of Cambridge, Strangeways Laboratory, Worts Causeway, Cambridge, UK, ^30^Fred Hutchinson Cancer Research Center, M4-B847, P.O. Box 19024, Seattle, WA 98109-1024, USA, ^31^Department of Epidemiology, Harvard School of Public Health, 677 Huntington Avenue, Boston, MA 02115, USA, ^32^Division of Epidemiology, Department of Medicine, Vanderbilt University Medical Center, 2525 West End Avenue, Suite 800, Nashville, TN 37232 USA, ^33^Mayo Clinic, Rochester, Minnesota, USA, ^34^Department of Urology, University Hospital Ulm, Germany, ^35^Department of Urology, Klinikum rechts der Isar der Technischen Universitaet Muenchen, Munich, Germany, ^36^Institute of Human Genetics, University Hospital Ulm, Germany, ^37^ International Epidemiology Institute, 1555 Research Blvd., Suite 550, Rockville, MD 20850, USA, ^38^International Hereditary Cancer Center, Department of Genetics and Pathology, Pomeranian Medical University, Szczecin, Poland, ^39^Division of Clinical Epidemiology and Aging Research, German Cancer Research Center (DKFZ), 69120 Heidelberg, Germany, ^40^ German Cancer Consortium (DKTK), 69120 Heidelberg, Germany, ^41^Saarland Cancer Registry, 66119 Saarbruecken, Germany, ^42^Division of Clinical Epidemiology and Aging Research, German Cancer Research Center (DKFZ), 69120 Heidelberg, Germany, ^43^Department of Cancer Epidemiology, Moffitt Cancer Center, 12902 Magnolia Drive, Tampa, FL 33612, USA, ^44^Biostatistics Program, Moffitt Cancer Center, 12902 Magnolia Drive, Tampa, FL 33612, USA, ^45^Department of General and Clinical Pathology, Medical University, Sofia, Bulgaria, ^46^ Department of Medical Chemistry and Biochemistry, Molecular Medicine Center, Medical University, Sofia, 2 Zdrave Str., 1431 Sofia, Bulgaria, ^47^Department of Urology and Alexandrovska University Hospital, Medical University, Sofia, Bulgaria, ^48^Australian Prostate Cancer Research Centre-Qld, Institute of Health and Biomedical Innovation and School of Biomedical Science, Queensland University of Technology, Brisbane, Australia, ^49^Molecular Cancer Epidemiology Laboratory, Queensland Institute of Medical Research, Brisbane, Australia, ^50^Department of Genetics, Portuguese Oncology Institute, Porto, Portugal, ^51^The University of Surrey, Guildford, Surrey, GU2 7XH, UK |
| --- |

**The UK Genetic Prostate Cancer Study Collaborators**

Mr Z Abbasi, Mr M Akhlil Abdul-Hamid, Mr Paul D Abel, Professor Paul H Abrams, Dr Fawzi A Adab, Mr Andrew Adamson, Mr A Adeyoju, Mr Naveed Afzal, Mr Ernest K N Ahiaku, Mr Munir Ahmed, Mr Mohammed L Al Sudani, Dr Christopher Alcock, Dr Zulfiqar Ali, Mr David J Almond, Dr Roberto Alonzi, Dr Amir S M Al-Samarraie, Dr Al-Samerraie, Mr Waleed Al-Singary, Mr Al-Sudani, Mr John Anderson, Mr Steven Andrews, Mr Henry Andrews, Mr Iqbal Anjum, Mr Ken Anson, Dr Nicola A Anyamene, Mr Ike Apakama, Dr F Aparcia, Mr J A A Archbold, Dr D Ash, Dr Richard F U Ashford, Dr A Azzabi, Mr David Badenoch, Dr Amit Bahl, Mr M J Bailey, Mrs Karen Bailey, Mr Andrew J Ball, Mr G Banerjee, Dr N Barber, Dr Jim Barber, Dr Baria, Mr Douglas G Barnes, Mr J Bashir, Mr Pradip Basu, Mr Christopher A Bates, Dr N A Bax, Mr D Baxter-Smith, Mr Amar Bdesha, Mr Christopher J M Beacock, Professor Ronald P Beaney, Mr Ralph Beard, Mr John D Beatty, Mr Rupert Beck, Ms Gail Beese, Dr Sharon Beesley, Mr C Richard W Bell, Mr James Bellringer, Dr Richard Benson, Dr Beresford, Mr Christopher R A Bevis, Dr Rajanee Bhana, Mr S Bhanot, Dr A Bhatnagar, Mr R I Bhatt, Mr Brian Birch, Dr Alison Birtle, Mr M Bishop, Mr C Shekhar Biyani, Mr A R E Blacklock, Miss Rosemary Blades, Dr Peter Bliss, Dr David J Bloomfield, Miss S Boddy, Professor C M Booth, Mr Pradeep Bose, Dr Michael C Bott, Dr David Bottomley, Mr Nigel R Boucher, Dr J Bowen, Dr Mark Bower, Mr W G Bowsher, Mr P J R Boyd, Mr F James Bramble, Mr Simon F Brewster, Mr Tim Briggs, Dr Cathryn Brock, Dr Sue Brock , Mr Stephen Bromage, Mr Richard Brough, Dr Richard Brown, Mr Stephen Brown, Mr Richard Brown, Mr Tony J Browning, Mr N Bryan, Mr Neil A Burgess, Mr Nicholas Burns-Cox, Mr Paul C Butterworth, Mr D Cahill, Mr P S Callaghan, Mr John Calleary, Dr M Calleja, Dr Frances Calman, Dr Philip Camilleri, Mr Alister Campbell, Miss Andrea Cannon, Dr Dawn M Carnell, Mr T W Carr, Mr Simon Carter, Mr Charles J M Carter, Dr Adam C Carter, Dr Bruce M Castle, Mr David Chadwick, Mr Rohit Chahal, Dr P Chakraborti, Mr Chappell, Mr C Charig, Dr Anula D Chetiyawardana, Mr Christopher Chilton, Mr F I Chinegwundoh, Dr Irene Chong, Dr Ananya Choudhury, Mr Wai-Man Chow, Mr Timothy J Christmas, Dr Mark J Churn, Mr Noel W Clarke, Mr Jorge Clavijo-Eisele, Dr M Coe, Mr N P Cohen, Mr C Coker, Dr Trevor Cole, Dr David J Cole, Mr O Cole, Mr Gerald Collins, Dr Matthew Collinson, Mr I Conn, Dr C Connell, Dr Audrey Cook, Mr Peter Cooke, Mr Graeme Cooksey, Mr L Coombs, Mr Robert F Copland, Mr Andrew J Cornaby, Mr P A Cornford, Mr Corolis, Mr John Corr, Mr C B Costello, Mrs N Coull, Dr Richard Cowan, Mr Robert Cox, Dr C Coyle, Mr Jeremy Crew, Mr John C Crisp, Dr W Cross, Mr W Cross, Dr Dorthe Cruger, Mr Malcolm Crundwell, Mr Cummings, Mr Nazeer Dahar, Dr Francis N Daniel, Mr J Darrad, Mr Pallon Daruwala, Mr Gautam Das, Mr Shibendra Datta, Dr S Davidson, Dr Joseph Davies, Mr Owen W Davison, Mr Guy Dawkins, Mr Chris Dawson, Mr Alan R De Bolla, Professor David Dearnaley, Mr Ken M Desai, Dr George P Deutsch, Mr John Dick, Mr Andrew J Dickinson, Dr Jeanette Dickson, Mr Michael Dinneen, Dr Sanjay Dixit, Dr H Jane Dobbs, Mr A Doble, Dr David Dodds, Mr Alan Doherty, Mr P Donaldson, Dr M Dooldeniya, Dr S Fiona Douglas, Mr Drake, Dr Gill M Duchesne, Mr Peter Duffy, Mr Michael Dunn, Mr W D Dunsmuir, Dr Sajid K Durrani, Mr Alan C Eaton, Professor Diane Eccles, Mr B Eddy, Mr C D Eden, Mr J Edwards, Mr Jeremy Elkabir, Dr P Tony Elliott, Mr B W Ellis, Dr R Ellis, Dr A El-Modir, Mr Andrew W S Elves, Dr Christine Elwell, Mr Mark Emberton, Dr Louise Emmerson, Mr Roland C D England, Mr R D Errington, Professor D Gareth Evans, Dr Alison Falconer, Mr Derek Fawcett, Dr C Featherston, Dr Carolyn J Featherstone, Mr Jeremy Feggetter, Dr C Ferguson, Dr D Fermont, Mr Michael Ferro, Mr Matthew Fletcher, Dr A Folkes, Mr Trevor F Ford, Mr Paul W Foster, Dr Kevin N Franks, Dr Olivera Frim, Dr Joanna Gale, Mr Christopher Gallegos, Mr James S Gelister, Dr Ghana, Dr Stephanie Gibbs, Mr Hugh Gilbert, Mr David Gillatt, Dr John Glaholm, Mr Jonathan M Glass, Mr James Glenister, Dr Thomas D Goode, Ms E M Gordon, Mr Richard L Gower, Dr John Graham, Mr Damian Green, Mr Jonathan Greenland, Dr Robert Grieve, Mr Thomas R L Griffiths, Mr Sandy Gujral, Dr Nishi Gupta, Mr Riza Murat Gurun, Mr Peter J Guy, Mr Neil Haldar, Mr N Halder, Professor F C Hamdy, Dr C Hamilton, Mr John Hammonds, Mr S J Hampson, Mr Damien C Hanbury, Dr P D John Hardman, Dr Stephen J Harland, Mr John M Harney, Dr Peter Harper, Dr Sarah Harris, Mr D Harris, Mr G S M Harrison, Mr D R Harriss, Mr N Harvey-Hills, Mr Simon Hawkyard, Dr Catherine M Heath, Mr Michael Hehir, Mr Giles O Hellawell, Mr David Hendry, Mr Mike Henley, Dr Ann Henry, Dr John Hetherington, Dr Tamas Hickish, Mr James A Hicks, Dr Serena Hilman, Mr Richard Hindley, Mr John R Hindmarsh, Mr John Hines, Dr M Hingorani, Mr Edwin T S Ho, Professor Shirley Hodgson, Dr U Hoffman, Mr David Holden, Dr A Hollingdale, Mr Graham W Hollins, Mr Simon A V Holmes, Dr Gail Horan, Professor Alan Horwich, Professor Peter Hoskin, Mr Graham P Howell, Mr D Hrouda, Dr Robert Huddart, Ms Liz Hudson, Dr Rob Hughes, Mr Michael Hughes, Mr Owen Hughes, Dr Caroline Humber, Mr John W Iacovou, Dr A Ibrahim, Mr John A Inglis, Mr Stuart Irving, Mr C Irwin, Dr Louise Izatt, Mr Victor Izegbu, Mr Basharat Jameel, Mr Michael J James, Professor N James, Mr R Lester James, Mr Pradip Javle, Dr P Jenkins, Dr Sameer Jhavar, Dr Gareth Jones, Mr Chris R Jones, Dr David A Jones, Mr J Joseph, Dr Shelagh Joss, Mr Amir Kaisary, Dr Alexandre L Kaliski, Dr G Kapur, Mr O Karim, Dr Stephen J Karp, Mr F X Keeley, Mr Anand R Kelkar, Mr J P Kelleher, Mr John Kelly, Dr Sue Kenwrick, Mr F Khan, Dr Vincent Khoo, Ms Rachel M Kimber, Mr R Kinder, Professor Roger S Kirby, Professor David Kirk, Dr Peter Kirkbride, Mr Magdi M Kirollos, Mr Roger Kockelbergh, Mr Philip C W C Koenig, Mr Gordon G Kooiman, Dr O Koreich, Mr Anthony Koupparis, Mr Mohamed Kourah, Dr Sigurd Kraus, Ms Magda L Kujawa, Mr Ravi Kulkarni, Mr M Kumar, Dr Ian H Kunkler, Professor H Kynaston, Dr Katherine L Lachlan, Dr Robert Laing, Dr Fiona Lalloo, Mr M Lancashire, Mr Stephen E M Langley, Mr Marc Laniado, Mr T R Larner, Mr Maurice W Lau, Mr W T Lawrence, Miss Anne Lawson, Mr Pieter J Le Roux, Professor Mary Leader, Mr J O Lee, Ms L Lee, Ms A Lee, Dr R John Lemburger, Dr Priscilla Leone, Dr Jason Lester, Mr Hing Leung, Mr J Lewis, Mr D Christopher Lewis, Mr Thomas Liston, Dr Jacqueline Livsey, Mr S Lloyd, Dr Imogen Locke, Mr Richard Lodge, Dr John Logue, Mr Mark Longmuir, Mr Malcolm G Lucas, Mr C J Luscombe, Dr Anna Lydon, Mr Michael Lynch, Mr Naing N K Lynn, Mr James P A MacDermott, Mr Ruaraidh P Macdonagh, Mr Macdonald, Mr Sanjeev Madaan, Dr Kudingila R Madhava, Dr Joseph Maguire, Professor E R Maher, Dr Rana Mahmood, Dr Graeme H M Mair, Mr Peter R Malone, Dr Stephen A Mangar, Mr Mark Mantle, Mr I Mark, Mr Robert Mason, Professor M D Mason, Mr Matanhelia, Mr Shyam Matenhelia, Mr Philip N Matthews, Dr J McAleese, Ms Donna McBride, Mr Jonathan McFarlane, Mr McGrath, Mr Craig McIlhenny, Mr Paul McInerney, Mr Gregor McIntosh, Dr F McKinna, Dr Duncan McLaren, Miss Esther McLarty, Dr Rhona McMenemin, Mr Alan McNeill, Mr T A McNicholas, Mr Robert N Meddings, Mr A David Mee, Dr Lucinda Melcher, Mr Memon, Mr Pravin Menzes, Mr Marek Miller, Mr Robert Mills, Mr S Mitchell, Dr Natasha Mithal, Dr Anita Mitra, Ms Gillian E Mobb, Mr Leslie E F Moffat, Mr Mokete, Dr Julian Money-Kyrle, Mr Bruce Montgomery, Mr Martin P Moody, Mr Roland Morley, Mr Sean B Morris, Professor Patrick Morrison, Dr Diana Mort, Mr Amir H Mostafid, Mr Hanif Motiwala, Mr Gulzar Mufti, Mr Gordon Muir, Mr Faiz Mumtaz, Mr Michael Murphy, Mr Keith W Murray, Dr Alexandra Murray, Dr Shirley Murrell, Dr D Muthukumar, Mr Harry Naerger, Mr Siva Namasivayam, Mr Vinod Nargund, Mr Nawrocki, Mr Donald Neilson, Dr A Nethersell, Mr Julian Barwell, Dr Jacqueline C Newby, Dr Hugh Newman, Dr R Newton, Mr Neil Oakley, Mr P J O'Boyle, Mr J O'Brien, Mr Tim S O'Brien, Dr H O'Donnell, Mr Neil O'Donoghue, Mr E O'Donoghue, Mr Chris Ogden, Mr Hemant Ohja, Professor Tim Oliver, Mr Eng K Ong, Mr P O'Reilly, Dr J S O'Rourke, Mr David Osborn, Dr Peter Ostler, Professor Joe O'Sullivan, Dr J Owen, Mr Edward Palfrey, Dr Miguel Panades, Dr Niki Panakis, Mr M Pancharatnam, Mr Michalakis L Pantelides, Dr U Panwar, Dr Omi Parikh, Dr Chris Parker, Mr Christopher H Parker, Mr Bohdan T Parys, Dr Sarah Pascoe, Mr Anup Patel, Dr Joan Paterson, Mr S Pathack, Ms Jhumur Pati, Dr Helen Patterson, Dr Pattu, Mr A Paul, Dr Heather Payne, Dr David Peake, Dr I Pedley, Mr A Pengelly, Mr Amjad M Peracha, Dr Matthew Perry, Mr Raj Persad, Mr John Peters, Mr N H Philp, Mr T Philp, Dr Lisa M Pickering, Dr Katharine Pigott, Mr R Plail, Dr P Nicholas Plowman, Mr Richard D Pocock, Mr A J Pope, Mr Rick Popert, Mr Tim Porter, Mr John M Potter, Mr Christopher Powell, Dr Thomas B Powles, Mr Krishna Prasad, Mr Seshadri Sri Prasad, Mr J W Prejbisz, Mr Stephen Prescott, Dr Andrew Protheroe, Mr Khaver N Qureshi, Dr Nigel Raby, Dr Narasimhan Ragavan, Mr Palaniappa G S Raju, Dr Prakash B Ramachandra, Dr R Raman, Mr Abhay Rane, Dr Julia Rankin, Mr Y Rao, Mr Hari L Ratan, Mr Ramachandran Ravi, Dr K Ravishankar, Dr Read, Mr Paul J Reddy, Mr Peter R Rimington, Dr Peter A Ritchie, Dr J Trevor Roberts, Mr Andrew Robertson, Dr Angus Robinson, Dr Anne C Robinson, Mr Lee Q Robinson, Mr Mark A Rochester, Mr P B Rogers, Mr Tomas P Rosenbaum, Mr Neil Rothwell, Mr Carl Rowbotham, Mr Rowe, Dr Kathryn Rowley, Dr Deborah Ruddy, Mr John Rundle, Dr John M Russell, Mr P G Ryan, Dr A Sabharwal, Dr Anand K Saggar, Dr Ali Samanci, Mr Vijay K Sangar, Mr M F Saxby, Mr Hartwig Schwaibold, Dr John E Scoble, Dr Christopher Scrase, Mr Selim, Mr Henry Sells, Mr Krishna K Sethia, Mr David C Shackley, Dr Shaffer, Dr Nihil Shah, Dr D Shakespeare, Dr Sue Shanley, Mr Neerah K Sharma, Dr Denise J Sheehan, Dr Elizabeth Sherwin, Dr Poh Lin Shum, Dr LucySide, Dr Norma Sidek, Professor Karol Sikora, Dr R Simcock, Mr Andrew M Sinclair, Mr Pravin Singh, Dr M Siva, Mr Michael F Smith, Mr James Smith, Dr Michael Sokal, Mr Graham M Sole, Mr Mark J Speakman, Dr Alexander Spiers, Dr Thiagarajan Sreenivasan, Dr Narayanan N Srihari, Mr Srinivasan, Mr Rajagopalan Sriram, Dr John N Staffurth, Dr D Stewart, Dr Andrew Stockdale, Mr Mark A Stott, Mr M J Stower, Mr John R Strachan, Professor Nicholas S A Stuart, Dr Elaine Sugden, Mr Duncan Summerton, Dr Santhanam Sundar, Mr S K Sundaram, Mr Gokarakonda Suresh, Mr Shabbir Susnerwala, Mr Kuchibhotla S Swami, Miss Stephanie J Symons, Dr Isabel Syndikus, Dr Saad Tahir, Dr J Tanquay, Dr John W Taylor, Dr J W Taylor, Mr T Terry, Dr Robert J Thomas, Mr Stephen A Thomas, Mr Alan Thompson, Dr Alastair H Thomson, Dr A Thurston, Dr Owen Tilsley, Mr Stuart F Tindall, Dr K Tipples, Dr Tong, Mr Hamid Toussi, Dr Elizabeth W Toy, Professor Richard C Trembath, Mr David N Tulloch, Mr Kevin J Turner, Mr James Tweedle, Dr C J Tyrell, Mr N Umez-Eronini, Mr Graeme H Urwin, Mr Justin A Vale, Dr Van As, Dr Nicholas Van As, Dr Subramaniam Vasanthan, Mr Sean Vesey, Dr Maria Vilarino-Varela, Dr John Violet, Mr Jaspal Virdi, Dr Robert Wade, Dr Katherine Waite, Mr E M Walker, Mr Roger Walker, Mr David M A Wallace, Mr Nicholas A Watkin, Mr M E Watson, Professor J H Waxman, Mr Brian Waymont, Dr Andrew Weaver, Mr Ralph J Webb, Mr Andrew Wedderburn, Dr Paula Wells, Mr G D Wemyss-Holden, Mr P M T Weston, Dr Duncan Wheatley, Mr P Whelan, Dr D Whillis, Mr Adam D Wilde, Dr Vicki Wiles, Dr Marie Wilkins, Mr John H Williams, Mr Simon Williams, Mr Michael Willis, Mr Michael I Wills, Mr Richard Wilson, Mr J R Wilson, Mr Mathias H Winkler, Dr Marcus Wise, Mr Simon Woodhams, Professor C Woodhouse, Dr Cathryn Woodward, Dr Woolf, Mr K A Woolfenden, Dr Jane Worlding, Mr Mark Wright, Dr WYLIE, Dr James P Wylie, Dr Chris Wynne, Ms Angelika Zang, Dr A Zarkar,

**The UK ProtecT Study Collaborators**

Angela Cox**,** Paul M. Brown**,** Anne George**,** Gemma Marsden**,** Athene Lane**,** Michael DavisPrasad Bollina, Sue Bonnington, Lynne Bradshaw, James Catto, Debbie Cooper, Liz Down, Andrew Doble, Alan Doherty, Garrett Durkan, Emma Elliott, David Gillatt, Pippa Herbert, Peter Holding, Joanne Howson, Mandy Jones, Roger Kockelbergh, Rajeev Kumar, Howard Kynaston, Athene Lane, Teresa Lennon, Norma Lyons, Hing Leung, Malcolm Mason, Hilary Moody, Philip Powell, Alan Paul, Stephen Prescott, Derek Rosario, Patricia O'Sullivan, Pauline Thompson, Sarah Tidball.

**Members of the PRACTICAL consortium by studies who are not listed as authors**

The Institute of Cancer Research & The Royal Marsden NHS Foundation Trust

Cyril Fisher, Charles Jameson

AUSTRALIA

Melbourne

Melissa C. Southey, John L. Hopper, Dallas R. English**,** John Pedersen

Queensland

Srilakshmi Srinivasan, Felicity Lose, Amanda Spurdle

Australian Prostate Cancer BioResource: Gail Risbridger, Wayne Tilley, Lisa Horvarth

Australian Prostate Cancer Bio Resource-QLD node: Peter Heathcote, Glenn Wood, Greg Malone, Hema Samaratunga, Pamela Saunders, Allison Eckert, Trina Yeadon, Kris Kerr, Angus Collins, Megan Turner

BULGARIA

PCMUS study

Medical University, Sofia,Department of Urology: Chavdar Slavov, Vanio Mitev, Elenko Popov

Molecular Medicine Center and Department of Chemistry and Biochemistry: Darina Kachakova, Atanaska Mitkova, Teodora Goranova, Gergana Stancheva, Olga Beltcheva, Rumyana Dodova

Department of General and Clinical Pathology: Aleksandrina Vlahova, Tihomir Dikov, Svetlana Christova

DENMARK

CPCS1

Department of Urology, Herlev Hospital, Copenhagen University Hospital,

Herlev, Denmark: Peter Klarskov, Børge G. Nordestgaard, M. Andreas Røder, Sune F. Nielsen, Stig E. Bojesen

CPCS2

Department of Urology, Rigshospitalet, Copenhagen University Hospital, Copenhagen, Denmark: Prof. DMSc Peter Iversen

FINLAND

University of Tampere and Tampere University Hospital, Tampere, Finland: Tiina Wahlfors, Teuvo LJ Tammela,Anssi Auvinen

The Finnish Cancer Registry, Helsinki, Finland: Liisa Määttänen

GERMANY

ESTHER

Saarland Cancer Registry, Saarbrücken, Germany: Christa Stegmaier

German Cancer Research Center (DKFZ), Heidelberg, Germany: Aida Karina Dieffenbach, Dietrich Rothenbacher

Ulm

Department of Urology, University Hospital Ulm, Germany: Walter Vogl, Antje E. Rinckleb**,** Manuel Luedeke**,** Mark Schrader

Institute of Human Genetics, University Hospital Ulm, Germany: Josef Hoegel

Department of Urology, Technical University Munich, Germany: Kathleen Herkommer

POLAND

Dominika Wokolorczyk, Jan Lubinski

PORTUGAL

IPO-Porto Study

Department of Pathology, Portuguese Oncology Institute, Porto, Portugal: Rui Henrique

Department of Genetics, Portuguese Oncology Institute, Porto, Portugal: Carmen Jerónimo, Pedro Pinto, Joana Santos, João D. Barros-Silva, Sofia Maia, Paula Paulo

SWEDEN

CAPS

Jan Adolfsson, Pär Stattin, Jan-Erik Johansson

STHM1

Carin Cavalli-Björkman, Ami Rönnberg Karlsson, Michael Broms

UNITED KINGDOM

The Institute of Cancer Research & The Royal Marsden NHS Foundation Trust, London

Cyril Fisher, Charles Jameson

EPIC-BPC3

Department of Epidemiology and Biostatistics, School of Public Health, Imperial College, London, United Kingdom: Hans Wallinder, Sven Gustafsson

UNITED STATES OF AMERICA

FHCRC

Fred Hutchinson Cancer Research Center, Seattle, US: Suzanne Kolb, Danielle M. Karyadi

MAYO

Shannon K McDonnell**,** Lori Tillmans**,** Shaun Riska

MEC-BPC3

Department of Preventive Medicine, Keck School of Medicine, University of Southern California, Los Angeles, CA, USA: Mariana C Stern, Roman Corral, Amit D. Joshi, Ahva Shahabi, Dan Stram

Epidemiology Program, University of Hawaii Cancer Center, Department of Medicine, John A. Burns School of Medicine, Univerisity of Hawaii, Honolulu, HI, USA: Kolonel Laurence

MOFFIT

Thomas A. Sellers, Hui-Yi Lin, Julio Pow-Sang, Hyun Y. Park, Selina Radlein, Maria Rincon

James A Haley VA Hospital, Tampa, FL, USA: Babu Zachariah

SALT LAKE CITY, UTAH

Robert A Stephenson, Craig Teerlink

**Details of additional funding**

The FHCRC, Mayo, MCCS, Tampere, UKGPCS and Ulm groups are part of the ICPCG supported by NIH Grant No. U01 CA089600-04.

The Prostate Cancer Program of Cancer Council Victoria also acknowledge grant support from The National Health and Medical Research Council, Australia (126402, 209057, 251533, 396414, 450104, 504700, 504702, 504715, 623204, 940394, 614296), VicHealth, Cancer Council Victoria, The Prostate Cancer Foundation of Australia, The Whitten Foundation, PricewaterhouseCoopers, and Tattersall’s. EAO, DMK, and EMK acknowledge the Intramural Program of the National Human Genome Research Institute for their support.

The QLD research is supported by The National Health and Medical Research Council, Australia Project Grant [390130, 1009458] and Enabling Grant [614296 to APCB]; the Prostate Cancer Foundation of Australia (Project Grant [PG7] and Research infrastructure grant [to APCB]).

We would like to acknowledge the support of The University of Cambridge, Cancer Research UK. Cancer Research UK grants [C8197/A10123] and [C8197/A10865] supported the genotyping team. We would also like to acknowledge the support of the National Institute for Health Research which funds the Cambridge Bio-medical Research Centre, Cambridge, UK. We would also like to acknowledge the support of the National Cancer Research Prostate Cancer: Mechanisms of Progression and Treatment (PROMPT) collaborative (grant code G0500966/75466) which has funded tissue and urine collections in Cambridge.

We are grateful to staff at the Welcome Trust Clinical Research Facility, Addenbrooke’s Clinical Research Centre, Cambridge, UK for their help in conducting the ProtecT study.  We also acknowledge the support of the NIHR Cambridge Biomedical Research Centre, the DOH HTA (ProtecT grant) and the NCRI / MRC (ProMPT grant) for help with the bio-repository. The UK Department of Health funded the ProtecT study through the NIHR Health Technology Assessment Programme (projects 96/20/06, 96/20/99). The ProtecT trial and its linked ProMPT and CAP (Comparison Arm for ProtecT) studies are supported by Department of Health, England; Cancer Research UK grant number C522/A8649, Medical Research Council of England grant number G0500966, ID 75466 and The NCRI, UK. The epidemiological data for ProtecT were generated though funding from the Southwest National Health Service Research and Development. DNA extraction in ProtecT was supported by USA Dept of Defense award W81XWH-04-1-0280, Yorkshire Cancer Research and Cancer Research UK. The authors would like to acknowledge the contribution of all members of the ProtecT study research group. The views and opinions expressed therein are those of the authors and do not necessarily reflect those of the Department of Health of England. The bio-repository from ProtecT is supported by the NCRI (ProMPT) Prostate Cancer Collaborative and the Cambridge BMRC grant from NIHR.

The Mayo group was supported by the US National Cancer Institute (R01CA72818).

The MEC was support by NIH grants CA63464, CA54281 and CA098758.

The Moffitt group was supported by the US National Cancer Institute (R01CA128813, PI: J.Y. Park).

The USC study was supported by the US National Cancer Institute (R01CA84979) and by the California Cancer Research Program (99-00524V-10258).

The San Francisco Bay Area Prostate Cancer Study was supported by the California Cancer Research Fund (99-00527V-10182).

The Tampere (Finland) study was supported by the Academy of Finland Grant 116437 and 251074, The Finnish Cancer Organisations, Sigrid Juselius Foundation, and The Medical Research Fund of Tampere University Hospital (# 9N069). The PSA screening samples were collected by the Finnish part of ERSPC ([European Study of Screening for Prostate Cancer](http://www.erspc.org/)).

The FHCRC studies were supported by grants RO1 CA056678, RO1 CA082664, and RO1 CA092579 from the National Cancer Institute, National Institutes of Health, with additional support from the Fred Hutchinson Cancer Research Center.

The Department of Medical Epidemiology and Biostatistics, Karolinska Institute, Stockholm, Sweden was supported by the Cancer Risk Prediction Center (CRisP; [www.crispcenter.org](http://www.crispcenter.org/)), a  Linneus Centre (Contract ID 70867902) financed by the Swedish Research Council, Swedish Research Council (grant no K2010-70X-20430-04-3), the Swedish Cancer Foundation (grant no 09-0677), the Hedlund Foundation, the Söderberg Foundation, the Enqvist Foundation, ALF funds from the Stockholm County Council. Stiftelsen Johanna Hagstrand och Sigfrid Linnér’s Minne, Karlsson’s Fund for urological and surgical research. We thank and acknowledge all of the participants in the Stockholm-1 study. We thank Carin Cavalli-Björkman and Ami Rönnberg Karlsson for their dedicated work in the collection of data. Michael Broms is acknowledged for his skilful work with the databases. KI Biobank is acknowledged for handling the samples and for DNA extraction. Hans Wallinder at Aleris Medilab and Sven Gustafsson at Karolinska University Laboratory are thanked for their good cooperation in providing historical laboratory results.

The PCMUS study was supported by the Bulgarian National Science Fund, Ministry of Education, Youth and Science (contract DOO-119/2009) with additional support from the Science Fund of Medical University - Sofia (contract 51/2009; 8I/2009).

SCCS is funded by NIH grant R01 CA092447, and SCCS sample preparation was conducted at the Epidemiology Biospecimen Core Lab that is supported in part by the Vanderbilt-Ingram Cancer Center (P30 CA68485). Data on SCCS cancer cases used in this publication were provided by the Alabama Statewide Cancer Registry; Kentucky Cancer Registry, Lexington, KY; Tennessee Department of Health, Office of Cancer Surveillance; Florida Cancer Data System; North Carolina Central Cancer Registry, North Carolina Division of Public Health; Georgia Comprehensive Cancer Registry; Louisiana Tumor Registry; Mississippi Cancer Registry; South Carolina Central Cancer Registry; Virginia Department of Health, Virginia Cancer Registry; Arkansas Department of Health, Cancer Registry, 4815 W. Markham, Little Rock, AR 72205. The Arkansas Central Cancer Registry is fully funded by a grant from National Program of Cancer Registries, Centers for Disease Control and Prevention (CDC). Data on SCCS cancer cases from Mississippi were collected by the Mississippi Cancer Registry which participates in the National Program of Cancer Registries (NPCR) of the Centers for Disease Control and Prevention (CDC). The contents of this publication are solely the responsibility of the authors and do not necessarily represent the official views of the CDC or the Mississippi Cancer Registry.

The Keith and Susan Warshaw Fund, C. S. Watkins Urologic Cancer Fund and The Tennity Family Fund supported the Utah study. The project was supported by Award Number P30CA042014 from the National Cancer Institute.

The ESTHER study was supported by a grant from the Baden-Württemberg Ministry of Science, Research and Arts.

The IPO-Porto study was in part supported by Liga Portuguesa Contra o Cancro and Fundação para a Ciência e a Tecnologia.

John Hopper is an Australia Fellow of the NHMRC. A. Spurdle is an NHMRC Senior Research Fellow, J. Clements is an NHMRC Principal Research Fellow, and J. Batra is an NHMRC Training Fellow. QLD study is currently supported by NHMRC grants APP1009458 and APP1050742.

The EuroBATS project was funded by the European Community’s Seventh Framework Programme (No. 259749) which also supports Ana Viñuela and Andrew A. Brown. Andrew A. Brown is also supported by a grant from the South-Eastern Norway Health Authority, (No. 2011060).

TwinsUK was funded by the Wellcome Trust; European Community’s Seventh Framework Programme (FP7/2007-2013). The study also receives support from the National Institute for Health Research (NIHR)- funded BioResource, Clinical Research Facility and Biomedical Research Centre based at Guy's and St Thomas' NHS Foundation Trust in partnership with King's College London. SNP Genotyping was performed by The Wellcome Trust Sanger Institute and National Eye Institute via NIH/CIDR.

**URLs**

<http://www.cancerresearchuk.org/cancer-info/cancerstats/types/prostate/incidence/>

[www.icr.ac.uk/ukgpcs](http://www.icr.ac.uk/ukgpcs)

<http://ec.europa.eu/research/health/medical-research/cancer/fp7-projects/cogs_en.html>

<http://ccge.medschl.cam.ac.uk/consortia/practical>

<http://mathgen.stats.ox.ac.uk/impute/data_download_1000G_phase1_integrated.html>

<http://pngu.mgh.harvard.edu/purcell/plink/>
